# Supplementary material for: Protocol for a scoping review to identify and map in-service education and training materials for midwifery care in sub-Saharan Africa from 2000 to 2020
Source: BMJ Open. 2021 Mar 24;11(3):e047118. doi: 10.1136/bmjopen-2020-047118 (PMC7993216; doi:10.1136/bmjopen-2020-047118)
Supplement: Supplementary data [file bmjopen-2020-047118supp003.pdf]

Supplementary file 3

Search strategy to be conducted in CINAHL

((((MH "Midwifery+") OR (MH "Students, Nurse Midwifery") OR (MH "Students, Midwifery") OR (MH "Lay Midwifery") OR (MH "Research, Midwifery") OR (MH "Nurse-Midwifery Service") OR (MH "Nurse Midwifery") OR (MH "Midwifery Service+")) AND ((MH "Professional Knowledge+") OR (MH "Clinical Competence+") OR (MH "Professional Competence+") OR (MH "Competency Assessment") OR (MH "Competency Assessment") OR (MH "Education, Competency-Based") OR (MH "Teaching Methods+/EV") OR (MH "Critical Thinking/EV")) AND (impact OR evaluation OR tools OR measuring OR exam* OR test* OR impact*)) OR (MH "Education, Midwifery" AND (evaluation OR impact OR tools OR testing OR exam* OR competen* OR "critical thinking"))

Welsh J, *et al. BMJ Open* 2021; 11:e047118. doi: 10.1136/bmjopen-2020-047118
