# Supplementary material for: Protocol for a scoping review to identify and map in-service education and training materials for midwifery care in sub-Saharan Africa from 2000 to 2020
Source: BMJ Open. 2021 Mar 24;11(3):e047118. doi: 10.1136/bmjopen-2020-047118 (PMC7993216; doi:10.1136/bmjopen-2020-047118)
Supplement: Supplementary data [file bmjopen-2020-047118supp004.pdf]

BMJ Publishing Group Limited (BMJ) disclaims all liability and responsibility arising from any reliance placed on this supplemental material which has been supplied by the author(s)

Supplementary file 4

Data extraction form

**Ref. ID**	**First Author**	**Year of Publication**	**Year(s) of Study Coverage**	**Study Design**	**Country**	**Study location**	**Study setting**	**Education area of focus**	**Cadres trained**	**On-site**	**Off-site**	**Online**	**Lectures**	**Simulation**	**Mentorship**	**Training Facilitator**	**Identifiable training materials**	

Welsh J, *et al. BMJ Open* 2021; 11:e047118. doi: 10.1136/bmjopen-2020-047118
