# Supplementary material for: Protocol for a scoping review to identify and map in-service education and training materials for midwifery care in sub-Saharan Africa from 2000 to 2020
Source: BMJ Open. 2021 Mar 24;11(3):e047118. doi: 10.1136/bmjopen-2020-047118 (PMC7993216; doi:10.1136/bmjopen-2020-047118)
Supplement: Supplementary data [file bmjopen-2020-047118supp005.pdf]

Supplementary file 5
Tools to map education and training material content to ICM Competencies for Midwifery Practice and Lancet Series Quality Maternal and Newborn Care framework

Data collection tool: Mapping to ICM Essential Competencies for Midwifery Practice

**Reference ID**									
**First author**									
**Year of publication**									
**Identified training resource**									
**Category 1: General Competencies**									
**1a: Assume responsibility for own decisions and actions as an autonomous practitioner**									
Knowledge	Principles of accountability and transparency								
	Principles and concepts of autonomy								
	Principles of self-assessment and reflective practice								
	Personal beliefs and their influence on practice								
	Knowledge of evidence-based practices								
Skills and Behaviours	Demonstrate behaviour that upholds the public trust in the profession								
	Participate in self-evaluation, peer review and other quality improvement activities								
	Balance the responsibility of the midwife to provide best care with the autonomy of the woman to make her own decisions								
	Explain the midwife's role in providing care that is based on relevant laws, ethics and evidence.								
**1b: Assume responsibility for self-care and self-development as a midwife**									
Knowledge	Strategies for managing personal safety particularly within the facility or community setting								
Skills and behaviours	Display skills in management of self in relation to time management, uncertainty, change and coping with stress								
	Assume responsibility for personal safety in various practice settings								
	Maintain up-to-date skills and knowledge concerning protocols, guidelines and safe practice								
	Remain current in practice by participating in continuing professional education for example, participating in learning opportunities that apply evidence to practice to improve care such as mortality reviews or policy reviews)								
	Identify and address limitations in personal skill, knowledge, or experience								
	Promote the profession of midwifery, including participation in professional organizations at the local and national level								
**1c: Appropriately delegate aspects of care and provide supervision**									
Knowledge	Policies and regulation related to delegation								
	Supportive strategies to supervise others								
	Role of midwives as preceptors, mentors, supervisors, and role models								
Skills and behaviours	Provide supervision to ensure that practice is aligned with evidence-based clinical practice guidelines								
	Support the profession's growth through participation in midwifery education in the roles of clinical preceptor, mentor, and role model								
**1d: Use research to inform practice**									
Knowledge	Principles of research and evidence-based practice								
	Epidemiologic concepts relevant to maternal and infant health								
	Global recommendations for practice and their evidence base (e.g. World Health Organization guidelines)								
Skills and behaviours	Discuss research findings with women and colleagues								

Welsh J, *et al. BMJ Open* 2021; 11:e047118. doi: 10.1136/bmjopen-2020-047118

Supplementary file 5

Tools to map education and training material content to ICM Competencies for Midwifery Practice and Lancet Series Quality Maternal and Newborn Care framework

	Support research in midwifery by participating in the conduct of research									
**1e: Uphold fundamental human rights of individuals when providing midwifery care**										
Knowledge	Laws and/or codes that protect human rights									
	Sexual, reproductive health rights of women and girls									
	Development of gender identity and sexual orientation									
	Principles of ethics and Human Rights within midwifery practice									
Skills and behaviours	Provide information to women about their sexual and reproductive health rights									
	Inform women about the scope of midwifery practice and women's rights and responsibilities									
	Provide information and support to individuals in complex situations where there are competing ethical principles and rights									
	Practice in accordance with philosophy and code of ethics of the ICM and national standards for health professionals									
	Provide gender sensitive care									
**1f: Adhere to jurisdictional laws, regulatory requirements, and codes of conduct for midwifery practice.**										
Knowledge	The laws and regulations of the jurisdiction regarding midwifery									
	National/state/local community standards of midwifery practice									
	Ethical principles									
	ICM and other midwifery philosophies, values, codes of ethics									
Skills and behaviours	Practise according to legal requirements and ethical principles									
	Meet requirements for maintenance of midwifery registration									
	Protect confidentiality of oral information and written records about care of women and infants									
	Maintain records of care in the manner required by the health authority									
	Comply with all local reporting regulations for birth and death registration									
	Recognize violations of laws, regulations, and ethical codes and take appropriate action									
	Report and document incidents and adverse outcomes as required while providing care									
**1g: Facilitate women to make individual choices about care**										
Knowledge	Cultural norms and practices surrounding sexuality, sexual practices, marriage, the childbearing continuum, and parenting									
	Principles of empowerment									
	Methods of conveying health information to individuals, groups, communities									
Skills and behaviours	Advocate for and support women to be the central decision makers in their care									
	Assist women to identify their needs, knowledge, skills, feelings, and preferences throughout the course of care									
	Provide information and anticipatory guidance about sexual and reproductive health to assist women's decision making									
	Collaborate with women in developing a comprehensive plan of care that respects her preferences and decisions									
**1h: Demonstrate effective interpersonal communication with women and families, health care teams, and community groups**										
Knowledge	Role and responsibilities of midwives and other maternal – infant health providers									
	Principles of effective communication									
	Principles of effectively working in health care teams									
	Cultural practices and beliefs related to childbearing and reproductive health									
	Principles of communication in crisis situations, e.g. grief and loss, emergencies									
Skills and behaviours	Listen to others in an unbiased and empathetic manner									

Supplementary file 5

Tools to map education and training material content to ICM Competencies for Midwifery Practice and Lancet Series Quality Maternal and Newborn Care framework

	Respect one others' point of view								
	Promote the expression of diverse opinions and perspectives								
	Use the preferred language of the woman or an interpreter to maximise communication								
	Establish ethical and culturally-appropriate boundaries between professional and non-professional relationships								
	Demonstrate cultural sensitivity to women, families, and communities								
	Demonstrate sensitivity and empathy for bereaved women and family members								
	Facilitate teamwork and inter-professional care with other care providers (including students) and community groups/ agencies								
	Establish and maintain collaborative relationships with individuals, agencies, institutions that are part of referral networks								
	Convey information accurately and clearly and respond to the needs of individuals								
**1i: Facilitate normal birth processes in institutional and community settings, including women's homes**									
Knowledge	Normal biologic, psychologic, social, and cultural aspects of reproduction and early life								
	Practices that facilitate and those that interfere with normal processes								
	Policies and protocols about care of women in institutional and community settings								
	Availability of resources in various settings								
	Community views about and utilization of health care facilities and place(s) of birth								
Skills and behaviours	Promote policies and a work culture that values normal birth processes								
	Utilize human and clinical care resources to provide personalized care for women and their infants								
	Provide continuity of care by midwives known to woman								
**1j: Assess the health status, screen for health risks, and promote general health and well-being of women and infants**									
Knowledge	Health needs of women related to reproduction								
	Health conditions that pose risks during reproduction								
	Health needs of infants and common risks								
Skills and behaviours	Conduct a comprehensive assessment of sexual and reproductive health needs								
	Assess risk factors and at-risk behaviour								
	Order, perform, and interpret laboratory and/ or imaging screening tests								
	Exhibit critical thinking and clinical reasoning informed by evidence when promoting health and well being								
	Provide health information and advice tailored to individual circumstances of women and their families								
	Collaborate with women to develop and implement a plan of care								
**1k: Prevent and treat common health problem related to reproduction and early life**									
Knowledge	Common health problems related to sexuality and reproduction								
	Common health problems and deviations from normal of newborn infants								
	Treatment of common health problems								
	Strategies to prevent and control the acquisition and transmission of environmental and communicable diseases								
Skills and behaviours	Maintain/promote safe and hygienic conditions for women and infants								
	Use universal precautions consistently								
	Provide options to women for coping with and treating common health problems								
	Use technology and interventions appropriately to promote health and prevent secondary complications								
	Recognize when consultation or referral is indicated for managing identified health problems, including consultation with other midwives								

Supplementary file 5

Tools to map education and training material content to ICM Competencies for Midwifery Practice and Lancet Series Quality Maternal and Newborn Care framework

	Include woman in decision-making about referral to other providers and services								
**1l: Recognise abnormalities and complications and institute appropriate treatment and referral**									
Knowledge	Complications/pathologic conditions related to health status								
	Emergency interventions/life-saving therapies								
	Limits of midwifery scope of practice and own experience								
	Available referral systems to access medical and other personnel to manage complications								
	Community/facility plans and protocols for accessing resources in timely manner								
Skills and behaviours	Maintain up-to-date knowledge, life-saving skills, and equipment for responding to emergency situations								
	Recognize situations requiring expertise beyond midwifery care								
	Maintain communication with women about nature of problem, actions taken, and referral if indicated								
	Determine the need for immediate intervention and respond appropriately								
	Implement timely and appropriate intervention, inter-professional consultation and/or timely referral taking account of local circumstances								
	Provide accurate oral and written information to other care providers when referral is made								
	Collaborate with decision-making if possible and appropriate								
**1m: Care for women who experience physical and sexual violence and abuse**									
Knowledge	Socio-cultural, behavioural, and economic conditions that often accompany violence and abuse								
	Resources in community to assist women and children								
	Risks of disclosure								
Skills and behaviours	Protect privacy and confidentiality								
	Provide information to all women about sources of help regardless of whether there is disclosure about violence								
	Inquire routinely about safety at home, at work								
	Recognize potential signs of abuse from physical appearance, emotional affect, related risk behaviours such as substance abuse								
	Provide special support for adolescents and victims of gender-based violence including rape								
	Refer to community resources, assist in locating safe setting as needed								
**Category 3: Care during labour and birth**									
**3a: Promote physiological labour and birth**									
Knowledge	Anatomy of maternal pelvis and fetus; mechanisms of labour for different fetal presentations								
	Physiologic onset and progression of labour								
	Evidence informed intrapartum care policies and guidelines, including avoidance of routine interventions in normal labour and birth								
	Cultural and social beliefs and traditions about birth								
	Signs and behaviours of labour progress; factors that impede labour progress								
	Methods of assessing fetus during labour								
Skills and behaviours	Provide care for a woman in the birth setting of her choice, following policies and protocols								
	Obtain relevant obstetric and medical history								
	Perform and interpret focused physical examination of the woman and fetus								
	Order and interpret laboratory tests if needed								
	Assess woman's physical and behavioural responses to labour								
	Provide information, support, and encouragement to woman and support persons throughoutlabour and birth								

Supplementary file 5

Tools to map education and training material content to ICM Competencies for Midwifery Practice and Lancet Series Quality Maternal and Newborn Care framework

	Provide respectful one-to-one care								
	Encourage freedom of movement and upright positions								
	Provide nourishment and fluids								
	Offer and support woman to use strategies for coping with labour pain, e.g. controlled breathing, water immersion, relaxation, massage, and pharmacologic modalities when needed								
	Assess regularly parameters of maternal-fetal status, and e.g. vital signs, contractions, cervical changes, and fetal descent								
	Use labour progress graphic display to record findings and assist in detecting complications, e.g. labour delay, fetal compromise, maternal exhaustion, hypertension, infection								
	Augment uterine contractility judiciously using non-pharmacological or pharmacological agents to prevent non-progressive labour								
	Prevent unnecessary routine interventions, e.g. amniotomy, electronic fetal monitoring, directed closed glottis pushing, episiotomy								
**3b: Manage a safe spontaneous vaginal birth; prevent, detect and stabilize complications**									
Knowledge	Manage a safe spontaneous vaginal birth								
	Evidence about conduct of third stage, including use of uterotonics								
	Potential complications and their immediate treatment e.g. shoulder dystocia,and excessive bleeding, fetal compromise, eclampsia, retained placenta								
	Management of emergencies as covered in emergency skills trainingprogrammes such as BEmONC,HMS								
	Signs of placental separation; appearance of normal placenta, membranes,and umbilical cord								
	Types of perineal and vaginal trauma requiring repair and suturing techniques								
Skills and behaviours	Support the woman to give birth in her position of choice								
	Ensure clean environment, presence of clean necessary supplies and sourceof warmth								
	Coach woman about pushing to control expulsion of presenting part, avoid routine episiotomy								
	Undertake appropriate manoeuvers and use maternal position to facilitatevertex, face, or breech birth								
	Expedite birth in presence of fetal distress								
	Delay cord clamping								
	Manage nuchal cord								
	Assess immediate condition of newborn								
	Provide skin to skin contact and warm environment								
	Deliver placenta and membranes and inspect for completeness								
	Assess uterine tone, maintain firm contraction, and estimate and record maternal blood loss; manage excessive blood loss including administration of uterotonics								
	Inspect vaginal and perineal areas for trauma, and repair as needed,following policies and protocols								
	Provide first line measures to treat or stabilise identified conditions								
	Refer for continuing treatment of any complications as needed								
**3c: Provide care of the newborn immediately after birth**									
Knowledge	Normal transition to extra-uterine environment								
	Scoring systems to assess newborn status								

5

Supplementary file 5
Tools to map education and training material content to ICM Competencies for Midwifery Practice and Lancet Series Quality Maternal and Newborn Care framework

	Signs indicating need for immediate actions to assist transition								
	Interventions to establish breathing and circulation as covered in training programs such as HBS								
	Appearance and behaviour of healthy newborn infant								
	Method of assessing gestational age of newborn infant								
	Needs of small for gestational age and low birth weight infants								
Skills and behaviour	Use standardized method to assess newborn condition in the first minutes of life (Apgar or other); refer if needed								
	Institute actions to establish and support breathing and oxygenation, refer for continuing treatment as needed								
	Provide a safe warm environment for initiating breastfeeding and attachment (bonding) in the first hour of life								
	Conduct a complete physical examination of newborn in presence of mother/family; explain findings and expected changes e.g. colour of extremities, moulding of head. Refer for abnormal findings.								
	Institute newborn prophylaxis e.g. ophthalmic infection, and hemorrhagic disease, according to policies and guidelines								
	Promote care by mother, frequent feeding and close observation								
	Involve partner/support persons in providing newborn care								

Supplementary file 5
Tools to map education and training material content to ICM Competencies for Midwifery Practice and Lancet Series Quality Maternal and Newborn Care framework

Data collection tool: Mapping to Lancet Series Quality Maternal and Newborn Care framework

**Reference ID**									
**First author**									
**Year of publication**									
**Identified training resource**									
**Practice Categories**									
For all childbearing women and infants	Education								
	Information								
	Health Promotion								
	Assessment								
	Screening								
	Care planning								
	Promotion of normal processes, prevention of complications								
For childbearing women and infants with complications	First line management of complications								
	Medical obstetric neonatal services								
**Organisation of care**									
For all childbearing women and infants AND for all childbearing women and infants with complications	Available								
	Accessible								
	Acceptable								
	Good quality services								
	Adequate resources								
	Competent workforce								
	Continuity								
	Services integrated across community and facilities								
**Values**									
For all childbearing women and infants AND for all childbearing women and infants with complications	Respect								
	Communication								
	Community knowledge								
	Understanding								
	Care tailored to women's circumstances and needs								
**Philosophy**									
For all childbearing women and infants AND for all childbearing women and infants with complications	Optimising biological, psychological, social and cultural processes								
	Strengthening women's capabilities								
	Expectant management, using interventions only when indicated								
**Care providers**									
For all childbearing women and infants AND for all childbearing women and infants with complications	Practitioners who combine clinical knowledge and skills with interpersonal and cultural competence								
	Division of roles and responsibility based on need, competencies and resources								

Welsh J, *et al. BMJ Open* 2021; 11:e047118. doi: 10.1136/bmjopen-2020-047118
