# Supplementary material for: Protocol for a scoping review to identify and map in-service education and training materials for midwifery care in sub-Saharan Africa from 2000 to 2020
Source: BMJ Open. 2021 Mar 24;11(3):e047118. doi: 10.1136/bmjopen-2020-047118 (PMC7993216; doi:10.1136/bmjopen-2020-047118)
Supplement: Author's manuscript [file bmjopen-2020-047118.draft_revisions.pdf]

BMJ Open

BMJ Open is committed to open peer review. As part of this commitment we make the peer review history of every article we publish publicly available.

When an article is published we post the peer reviewers' comments and the authors' responses online. We also post the versions of the paper that were used during peer review. These are the versions that the peer review comments apply to.

The versions of the paper that follow are the versions that were submitted during the peer review process. They are not the versions of record or the final published versions. They should not be cited or distributed as the published version of this manuscript.

BMJ Open is an open access journal and the full, final, typeset and author-corrected version of record of the manuscript is available on our site with no access controls, subscription charges or pay-per-view fees (http://bmjopen.bmj.com).

If you have any questions on BMJ Open's open peer review process please email info.bmjopen@bmj.com

BMJ Open

Protocol for a scoping review to identify and map in-service education and training materials for midwifery care in sub-Saharan Africa from 2000-2020

Journal:	*BMJ Open*
Manuscript ID	bmjopen-2020-047118
Article Type:	Protocol
Date Submitted by the Author:	21-Nov-2020
Complete List of Authors:	Welsh, Joanne; Hannover Medical School, Midwifery Research and Education Unit<br>Gross, MM; Hannover Medical School, Midwifery Research and Education Unit<br>Hanson, Claudia; Karolinska Institute, Dept Public Global Health<br>Hounkpatin , Hashim; Centre de Recherche en Reproduction Humaine et en Démographie (CERRHUD),<br>Moller, Ann-Beth; University of Gothenburg Institute of Medicine,
Keywords:	OBSTETRICS, PUBLIC HEALTH, REPRODUCTIVE MEDICINE

SCHOLARONE™
Manuscripts

I, the Submitting Author has the right to grant and does grant on behalf of all authors of the Work (as defined in the below author licence), an exclusive licence and/or a non-exclusive licence for contributions from authors who are: i) UK Crown employees; ii) where BMJ has agreed a CC-BY licence shall apply, and/or iii) in accordance with the terms applicable for US Federal Government officers or employees acting as part of their official duties; on a worldwide, perpetual, irrevocable, royalty-free basis to BMJ Publishing Group Ltd ("BMJ") its licensees and where the relevant Journal is co-owned by BMJ to the co-owners of the Journal, to publish the Work in this journal and any other BMJ products and to exploit all rights, as set out in our licence.

The Submitting Author accepts and understands that any supply made under these terms is made by BMJ to the Submitting Author unless you are acting as an employee on behalf of your employer or a postgraduate student of an affiliated institution which is paying any applicable article publishing charge ("APC") for Open Access articles. Where the Submitting Author wishes to make the Work available on an Open Access basis (and intends to pay the relevant APC), the terms of reuse of such Open Access shall be governed by a Creative Commons licence – details of these licences and which Creative Commons licence will apply to this Work are set out in our licence referred to above.

Other than as permitted in any relevant BMJ Author's Self Archiving Policies, I confirm this Work has not been accepted for publication elsewhere, is not being considered for publication elsewhere and does not duplicate material already published. I confirm all authors consent to publication of this Work and authorise the granting of this licence.

For peer review only

Protocol for a scoping review to identify and map in-service education and training materials for midwifery care in sub-Saharan Africa from 2000-2020

Author names

Joanne Welsh[1], Mechthild M. Gross[1], Claudia Hanson[2,3], Hashim Hounkpatin[4], Ann-Beth Moller[5].

1. Midwifery Research and Education Unit, Hannover Medical School Hannover. Hannover, Germany.

2. Global Public Health, Karolinska Institute. Stockholm, Sweden.

3. Department of Disease Control. London School of Hygiene and Tropical Medicine. London, United Kingdom.

4. Centre de Recherche en Reproduction Humaine et en Démographie (CERRHUD). Cotonou, Benin.

5. School of Public Health and Community Medicine, Institute of Medicine, University of Gothenburg, Gothenburg, Sweden.

Corresponding author: Ann-Beth Moller

School of Public Health and Community Medicine, Institute of Medicine, University of Gothenburg. Box 100, SE-405 30, Gothenburg, Sweden.

Email: ann-beth.moller.2@gu.se

Word Count: 1849 words

For peer review only - http://bmjopen.bmj.com/site/about/guidelines.xhtml

Abstract

Introduction

Maternal and neonatal mortality are disproportionally high in low- and middle-income countries. In 2017 the global maternal mortality ratio (MMR) was estimated to be 211 per 100,000 live births. An estimated 66% of these deaths occurred in sub-Saharan Africa. Training programmes that aim to prepare providers of midwifery care vary considerably across sub-Saharan Africa in terms of length, content and quality. To overcome the shortfalls of pre-service training and support the provision of quality care, in-service training packages for providers of midwifery care have been developed and implemented in many countries in sub-Saharan Africa. We aim to identify what in-service education and training materials have been used for providers of midwifery care between 2000-2020 and map their content to the International Confederation of Midwives' Essential Competencies for Midwifery Practice (ICM Competencies), and the Lancet Midwifery Series Quality Maternal and Newborn Care (QMNC) Framework.

Methods and analysis

A search will be conducted for the years 2000-2020 in Cumulative Index of Nursing and Allied Health Literature (CINAHL), African Index Medicus and Google Scholar. A manual search of reference lists from identified studies and a hand search of literature from international partner organisations will be performed. Information retrieved will include study context, providers trained, focus of training, and design of training. Original content of identified education and training materials will be obtained and mapped to the ICM Competencies and the Lancet Series QMNC.

For peer review only

For peer review only - http://bmjopen.bmj.com/site/about/guidelines.xhtml

Ethics and dissemination

A scoping review is a secondary analysis of published literature and does not require ethical approval. This scoping review will give an overview of the education and training materials used for in-service training for providers of midwifery care in sub-Saharan Africa. Mapping the content of these education and training materials to the ICM Competencies and The Lancet Series QMNC will allow us to assess their appropriateness. Findings from the review will be reflected to stakeholders involved in the design and implementation of such materials. Additionally, findings will be used to inform the design and content of an in-service training package for providers of midwifery care as part of the Action Leveraging Evidence to Reduce perinatal morTality and morbidity (ALERT) study, (https://alert.ki.se/) a multi country study in Benin, Malawi, Tanzania and Uganda.

Strengths and limitations of this study

- This scoping review will give a comprehensive overview of the education and training materials used for in-service training for providers of midwifery care in sub-Saharan Africa.
- To our knowledge this is the first review that aims to map the content of in-service education and training materials used for providers of midwifery care in sub-Saharan Africa to the ICM Essential Competencies and The Lancet Series Quality Maternal and Newborn Care Framework.
- This scoping review may miss in-service training materials that have not been reported in journals or published in the grey literature.

INTRODUCTION

A transformative agenda for maternal and newborn health has been laid out as part of the Sustainable Development Goals (SDGs); “to reduce the global maternal mortality ratio (MMR) to less than 70 per 100 000 live births by 2030” (SDG 3.1.) and “end preventable deaths of newborns, with all countries aiming

For peer review only - http://bmjopen.bmj.com/site/about/guidelines.xhtml

to reduce neonatal mortality to at least as low as 12 per 1000 live births" (SDG 3.2).[1] Maternal and neonatal mortality are disproportionally high in low- and middle-income countries (LMICs). In 2017 the global maternal mortality ratio (MMR) was estimated to be 211 per 100,000 live births. An estimated 66% of these deaths occurred in sub-Saharan Africa, whose regional MMR in 2017 was 542.[2] Similarly, despite a global neonatal mortality rate of 17 per 1,000 live births in 2019 this rate in sub-Saharan Africa was 27 per 1,000.[3] With access to evidence based, quality care the majority of these deaths are preventable. Within an enabling environment, midwifery care is effective in improving care and supports the international agenda to end preventable maternal and newborn deaths. [4 5] While today many more women give birth with a skilled birth attendant,[6] mortality reductions have been less than anticipated.[7] This paradox might be explained in relation to the complexity of provision of high-quality maternity care which is reliant on several factors including an appropriately trained and qualified health workforce who are equitably distributed, an enabling environment which includes access to essential equipment, medications and supplies as well as social and cultural aspects of care.

The workforce providing midwifery care is diverse. A study by Hobbs et al.[8] identified 102 unique cadre names from 36 LMICs who were involved in the provision of pregnancy, childbirth and postpartum care. For this review the term "provider of midwifery care" is used. See box 1. for the definition of provider of midwifery care used in this review.

<u>Definition of provider of midwifery care</u>

Providers of midwifery care are competent maternal and newborn health professionals educated, trained and regulated to national and/or international standards. They provide skilled, evidence-based and compassionate care to women, newborns and families.

Providers of midwifery care:

- Promote and facilitate normal physiological, social and cultural processes throughout the childbearing continuum with a continuity of care philosophy;
- Seek to prevent and manage maternal and newborn complications;
- Consult and refer to other health services when required; and
- Respect women's individual circumstances and views, providing sensitive and dignified care.

For peer review only - http://bmjopen.bmj.com/site/about/guidelines.xhtml

Box 1: Definition of midwifery care [9, 10]

Substantial heterogeneity exists in the length, content and quality of training that different providers of midwifery care receive to prepare them for the workforce, as well as in their ability to perform obstetric and neonatal functions. [8, 11] Inadequate training has been found to diminish the competence and confidence of providers of midwifery care.[12] In-service education therefore plays an important role in filling gaps that arise from inadequate pre-service education. It also acts to support continuous professional development for providers of midwifery care. A number of in-service training packages for providers of midwifery care have been developed and implemented in sub-Saharan Africa as a means to support the provision of quality maternity care.[13-16] Many in-service training packages focus on emergency obstetric care,[13-17] with few focusing on normal childbirth,[18] and dignified and respectful care.[19]

It is imperative that in-service education is evidence based, of high quality and promotes both autonomy and multidisciplinary team working. The International Confederation of Midwives[20] "Essential Competencies for Midwifery Practice" outline the minimum set of knowledge, skills and professional behaviour expected of an individual completing their midwifery training and joining the workforce. These essential competencies are helpful in the design and evaluation of ongoing in-service training for providers of midwifery care. Additionally, the Lancet Series Quality Maternal and Newborn Care (QMNC) framework describes the components of care that are critical to the provision of best quality maternity care and places women's views and experiences at its core.[9] The framework identifies effective practices, the organization of care, the philosophy and values of care providers and the characteristics of care providers as essential components to the provision of quality care. The framework is designed to be relevant to any setting and as such has been recommended as a structure around which improvements in midwifery can be made globally.[21]

For peer review only

For peer review only - http://bmjopen.bmj.com/site/about/guidelines.xhtml

This review is a component of the Action Leveraging Evidence to Reduce perinatal morTality and morbidity in sub-Saharan Africa (ALERT) project (Trial registration number: PACTR202006793783148).[22] The objective of the ALERT project is to develop and evaluate a multifaceted intervention to i) strengthen the implementation of evidence-based interventions and responsive care and, ii) reduce in-facility perinatal mortality and morbidity through a multidisciplinary approach in Benin, Malawi, Tanzania and Uganda. To achieve this, the ALERT project aims to develop and implement a co-designed in-service midwifery training package with a focus on basic intrapartum care. To inform this process this review aims to identify and map in-service education and training materials for providers of midwifery care that are available and have been used in sub-Saharan Africa since 2000.

METHODS AND ANALYSIS

Patient and Public Involvement

No patient involved

Study design

Due to the nature of the research question a scoping review design was chosen to allow for identification and mapping of existing in-service education and training materials. This scoping review follows the steps as outlined by Arksey and O'Malley.[23] The Preferred Reporting Items for Systematic reviews and Meta-Analyses extension for Scoping Reviews (PRISMA-ScR) Checklist (see supplementary file 1) will be used to present the findings of the scoping review, and the PRISMA flow diagram (see supplementary file 2) will be used to map out the phases of literature identification; the number of records identified, and those included and excluded with reasons given for exclusions.[24]

Research questions

For peer review only - http://bmjopen.bmj.com/site/about/guidelines.xhtml

The objectives of our study are to identify, and map in-service education and training materials designed for providers of midwifery care in sub-Saharan Africa from 2000-2020. The scoping review aims to address the following questions:

1. What in-service education and training materials are available and have been used for providers of midwifery care in sub-Saharan Africa from 2000-2020?
2. How does the content of these in-service education and training materials align with the International Confederation of Midwives Essential Competencies for Midwifery Practice as well as in relation to the Lancet Midwifery Series QMNC Framework?

Inclusion criteria

The research questions will be assessed, and studies will be selected specific to the following Population, Concept, Study Design and Context criteria presented in Table 1. All studies and grey literature that include information on available in-service education and training materials used for providers of maternity care in sub-Saharan Africa will be included in the review.

Table 1. Inclusion criteria for identifying eligible studies

	**Inclusion Criteria**
**Population**	Any in-service training on midwifery care aimed at any health professional who provides midwifery care.
**Concept**	Mapping the content of in-service training materials used for providers of midwifery care to the ICM Essential Competencies and The Lancet Series QMNC Framework.
**Study Design**	Studies and reports/documents that report on the implementation of in-service training materials and in-service training resources identified through searches of grey literature.
**Context**	In-service education in sub-Saharan Africa.

Exclusion criteria

Articles will not be eligible for inclusion in the scoping review if:

For peer review only - http://bmjopen.bmj.com/site/about/guidelines.xhtml

1. The country in which the in-service education and training has taken place is not located within sub-Saharan Africa.
2. The health personnel who have undertaken the in-service education and training is not considered to be a provider of midwifery care.
3. The content of the in-service education and training does not relate to the provision of midwifery care.
4. There are no details provided on the content of the education and training sessions.

Search strategy

The search strategy will be conducted for all relevant existing literature, without language restrictions, based on search terms relating to the research questions and restricted to the years 2000-2020 using the following online bibliographic databases: Cumulative Index of Nursing and Allied Health Literature (CINAHL), African Index Medicus, and Google Scholar. Grey literature searches will be performed and include organizations known to be active in global health improvement (i.e. United Nations Population Fund [UNFPA], World Health Organization [WHO], Johns Hopkins Program for International Education in Gynecology and Obstetrics [JHPIEGO], International Confederation of Midwives [ICM], International Federation of Gynecology and Obstetrics [FIGO], International Pediatric Association [IPA]). The reference list of all eligible studies will be hand-searched to identify any additional relevant studies. Examples of the preliminary search terms and strategy to be conducted in CINAHL are outlined in supplementary file 3.

Study selection

Following the aforementioned comprehensive search strategy, article titles and abstracts will be screened and eligibility for inclusion assessed independently by two reviewers. Any disagreements will

For peer review only

For peer review only - http://bmjopen.bmj.com/site/about/guidelines.xhtml

be resolved by a third reviewer. Screened abstracts identified for inclusion will undergo a full text review. Full text articles that are excluded at the screening stage will have reasons for exclusion documented. The Covidence[25] software will be used for both title/abstract and full text screening.

Data extraction and analysis

A data collection tool will be used to extract required information (see supplementary file 4). Information retrieved will include study context (country and health care setting). Details of the cadres of providers of midwifery care trained will be recorded. Further data will be collected regarding the focus of the training and design of the training (formal education sessions, training facilitators, on-site training, off-site training, use of simulation, ongoing mentorship, online training). The data extraction form will contain additional fields to allow for flexibility should any other categories of training design arise that the authors had not initially considered.

Where possible, original content of identified education and training materials will be obtained. This will allow original content to be mapped to the ICM Essential Competencies for Midwifery Practice[20] and the Lancet Series QMNC[9]. The tools used to map content of education and training materials can be found in supplementary file 5. As the scoping review aims to map the content of in-service training materials we will not assess the quality of the studies reporting on the in-service training. All studies meeting inclusion criteria will therefore be included in the review. Descriptive statistics will be used to summarise the findings on how the identified in-service training materials contain content pertaining to the ICM Essential Competencies and the Lancet Series QMNC framework.

DISSEMINATION AND ETHICS

For peer review only - http://bmjopen.bmj.com/site/about/guidelines.xhtml

This scoping review will give a comprehensive overview of the education and training materials used for in-service training of providers of midwifery care in sub-Saharan Africa. To our knowledge this is the first review that aims to map the content of education and training materials used for providers of midwifery care in sub-Saharan Africa to the ICM Essential Competencies and The Lancet Series QMNC framework. To ensure future in-service education and training materials are of quality and designed to adhere to the ICM Essential Competencies and the Lancet Series QMNC framework, findings from the scoping review will be reflected to stakeholders involved in the design and implementation of such materials and will be used to inform the development of the ALERT in-service midwifery training package. Scoping reviews collect and examine data from existing literature and therefore do not require prior ethical approval.

Author's contributions

JW wrote the original draft. ABM contributed to the conception of the study and substantively revised the manuscript. JW and ABM developed the data extraction form. All authors have contributed to the manuscript and read and approved the final version.

Funding statement

This study is part of the ALERT-project which is funded by the European Commission's Horizon 2020 (No 847824) under a call for Implementation research for maternal and child health. The contents of this article are solely the responsibility of the authors and do not reflect the views of the EU.

Competing Interests

The authors declare that they have no competing interests.

Supplementary files

Supplementary file 1: The Preferred Reporting Items for Systematic reviews and Meta-Analyses extension for Scoping Reviews (PRISMA-ScR) Checklist.

For peer review only

For peer review only - http://bmjopen.bmj.com/site/about/guidelines.xhtml

Supplementary file 2: The Preferred Reporting Items for Systematic reviews and Meta-Analyses (PRISMA) flow diagram.

Supplementary file 3: Preliminary search terms and strategy to be conducted in CINAHL.

Supplementary file 4: Data extraction form.

Supplementary file 5: Tools to map education and training material content to ICM Competencies for Midwifery Practice and Lancet Series Quality Maternal and Newborn Care framework.

References

1. Transforming our world: the 2030 Agenda for Sustainable Development. Resolution adopted by the General Assembly on 25 September 2015. New York (NY): United Nations, 2015.
2. WHO, UNICEF, UNFPA, et al. Trends in maternal mortality: 2000 to 2017: estimates by WHO, UNICEF, UNFPA, The World Bank Group and the United Nations Population Division. Geneva: World Health Organization, 2019.
3. United Nations Inter-agency Group for Child Mortality Estimation. Levels and Trends in Child Mortality: Report 2020, Estimates developed by the United Nations Inter-agency Group for Child Mortality Estimation. New York, 2020.
4. Homer CS, Friberg IK, Dias MA, et al. The projected effect of scaling up midwifery. *Lancet* 2014;384(9948):1146-57. doi: 10.1016/s0140-6736(14)60790-x [published Online First: 2014/06/27]
5. Sandall J, Soltani H, Gates S, et al. Midwife-led continuity models versus other models of care for childbearing women. *Cochrane Database Syst Rev* 2016;4:Cd004667. doi: 10.1002/14651858.CD004667.pub5 [published Online First: 2016/04/29]
6. Montagu D, Sudhinaraset M, Diamond-Smith N, et al. Where women go to deliver: understanding the changing landscape of childbirth in Africa and Asia. *Health Policy Plan* 2017;32(8):1146-52. doi: 10.1093/heapol/czx060 [published Online First: 2017/05/26]
7. Ng M, Misra A, Diwan V, et al. An assessment of the impact of the JSY cash transfer program on maternal mortality reduction in Madhya Pradesh, India. *Glob Health Action* 2014;7:24939. doi: 10.3402/gha.v7.24939 [published Online First: 2014/12/06]
8. Hobbs AJ, Moller AB, Kachikis A, et al. Scoping review to identify and map the health personnel considered skilled birth attendants in low-and-middle income countries from 2000-2015. *PLoS One* 2019;14(2):e0211576. doi: 10.1371/journal.pone.0211576 [published Online First: 2019/02/02]
9. Renfrew MJ, McFadden A, Bastos MH, et al. Midwifery and quality care: findings from a new evidence-informed framework for maternal and newborn care. *Lancet* 2014;384(9948):1129-45. doi: 10.1016/s0140-6736(14)60789-3 [published Online First: 2014/06/27]
10. World Health Organization. Defining competent maternal and newborn health professionals. Geneva, 2018.

For peer review only - http://bmjopen.bmj.com/site/about/guidelines.xhtml

11. Michel-Schuldt M, McFadden A, Renfrew M, et al. The provision of midwife-led care in low-and middle-income countries: An integrative review. *Midwifery* 2020;84:102659. doi: 10.1016/j.midw.2020.102659 [published Online First: 2020/02/18]
12. Filby A, McConville F, Portela A. What Prevents Quality Midwifery Care? A Systematic Mapping of Barriers in Low and Middle Income Countries from the Provider Perspective. *PLoS One* 2016;11(5):e0153391. doi: 10.1371/journal.pone.0153391 [published Online First: 2016/05/03]
13. Evans CL, Bazant E, Atukunda I, et al. Peer-assisted learning after onsite, low-dose, high-frequency training and practice on simulators to prevent and treat postpartum hemorrhage and neonatal asphyxia: A pragmatic trial in 12 districts in Uganda. *PLoS One* 2018;13(12):e0207909. doi: 10.1371/journal.pone.0207909 [published Online First: 2018/12/18]
14. Egenberg S, Masenga G, Bru LE, et al. Impact of multi-professional, scenario-based training on postpartum hemorrhage in Tanzania: a quasi-experimental, pre- vs. post-intervention study. *BMC Pregnancy Childbirth* 2017;17(1):287. doi: 10.1186/s12884-017-1478-2 [published Online First: 2017/09/07]
15. Ameh CA, Kerr R, Madaj B, et al. Knowledge and Skills of Healthcare Providers in Sub-Saharan Africa and Asia before and after Competency-Based Training in Emergency Obstetric and Early Newborn Care. *PLoS One* 2016;11(12):e0167270. doi: 10.1371/journal.pone.0167270 [published Online First: 2016/12/23]
16. Crofts JF, Mukuli T, Murove BT, et al. Onsite training of doctors, midwives and nurses in obstetric emergencies, Zimbabwe. *Bull World Health Organ* 2015;93(5):347-51. doi: 10.2471/blt.14.145532 [published Online First: 2015/08/01]
17. Gavine A, MacGillivray S, McConville F, et al. Pre-service and in-service education and training for maternal and newborn care providers in low- and middle-income countries: An evidence review and gap analysis. *Midwifery* 2019;78:104-13. doi: 10.1016/j.midw.2019.08.007 [published Online First: 2019/08/17]
18. Makowiecka K, Achadi E, Izati Y, et al. Midwifery provision in two districts in Indonesia: how well are rural areas served? *Health Policy Plan* 2008;23(1):67-75. doi: 10.1093/heapol/czm036 [published Online First: 2007/11/01]
19. Fujita N, Perrin XR, Vodounon JA, et al. Humanised care and a change in practice in a hospital in Benin. *Midwifery* 2012;28(4):481-8. doi: 10.1016/j.midw.2011.07.003 [published Online First: 2011/09/20]
20. The International Confederation of Midwives. The International Confederation of Midwives (ICM) Essential Competencies for Midwifery Practice Framework 2019.
21. Bharj KK, Luyben A, Avery MD, et al. An agenda for midwifery education: Advancing the state of the world's midwifery. *Midwifery* 2016;33:3-6. doi: 10.1016/j.midw.2016.01.004 [published Online First: 2016/01/27]
22. Action Leveraging Evidence to reduce perinatal Mortality and morbidity in sub-Saharan Africa project (ALERT) Sweden: Karolinska Institute; 2020 [Available from: https://ki.se/en/gph/the-alert-intervention-research-project accessed 13 April 2020.
23. Arksey H OML. Scoping studies: towards a methodological framework. *Int J Soc Res Methodol* 2005(8):19-32.
24. Liberati A, Altman DG, Tetzlaff J, et al. The PRISMA statement for reporting systematic reviews and meta-analyses of studies that evaluate healthcare interventions: explanation and elaboration. *BMJ* 2009;339:b2700. doi: 10.1136/bmj.b2700 [published Online First: 2009/07/23]
25. Covidence 2020 [Available from: https://www.covidence.org/].

For peer review only

Supplementary file 1: Preferred Reporting Items for Systematic reviews and Meta-Analyses extension for Scoping Reviews (PRISMA-ScR) Checklist

SECTION	ITEM	PRISMA-ScR CHECKLIST ITEM	REPORTED ON PAGE #
**TITLE**			
Title	1	Identify the report as a scoping review.	Click here to enter text.
**ABSTRACT**			
Structured summary	2	Provide a structured summary that includes (as applicable): background, objectives, eligibility criteria, sources of evidence, charting methods, results, and conclusions that relate to the review questions and objectives.	Click here to enter text.
**INTRODUCTION**			
Rationale	3	Describe the rationale for the review in the context of what is already known. Explain why the review questions/objectives lend themselves to a scoping review approach.	Click here to enter text.
Objectives	4	Provide an explicit statement of the questions and objectives being addressed with reference to their key elements (e.g., population or participants, concepts, and context) or other relevant key elements used to conceptualize the review questions and/or objectives.	Click here to enter text.
**METHODS**			
Protocol and registration	5	Indicate whether a review protocol exists; state if and where it can be accessed (e.g., a Web address); and if available, provide registration information, including the registration number.	Click here to enter text.
Eligibility criteria	6	Specify characteristics of the sources of evidence used as eligibility criteria (e.g., years considered, language, and publication status), and provide a rationale.	Click here to enter text.
Information sources*	7	Describe all information sources in the search (e.g., databases with dates of coverage and contact with authors to identify additional sources), as well as the date the most recent search was executed.	Click here to enter text.
Search	8	Present the full electronic search strategy for at least 1 database, including any limits used, such that it could be repeated.	Click here to enter text.
Selection of sources of evidence†	9	State the process for selecting sources of evidence (i.e., screening and eligibility) included in the scoping review.	Click here to enter text.
Data charting process‡	10	Describe the methods of charting data from the included sources of evidence (e.g., calibrated forms or forms that have been tested by the team before their use, and whether data charting was done independently or in duplicate) and any processes for obtaining and confirming data from investigators.	Click here to enter text.
Data items	11	List and define all variables for which data were sought and any assumptions and simplifications made.	Click here to enter text.
Critical appraisal of individual sources of evidence§	12	If done, provide a rationale for conducting a critical appraisal of included sources of evidence; describe the methods used and how this information was used in any data synthesis (if appropriate).	Click here to enter text.

For peer review only - http://bmjopen.bmj.com/site/about/guidelines.xhtml

SECTION	ITEM	PRISMA-ScR CHECKLIST ITEM	REPORTED ON PAGE #
Synthesis of results	13	Describe the methods of handling and summarizing the data that were charted.	Click here to enter text.
**RESULTS**			
Selection of sources of evidence	14	Give numbers of sources of evidence screened, assessed for eligibility, and included in the review, with reasons for exclusions at each stage, ideally using a flow diagram.	Click here to enter text.
Characteristics of sources of evidence	15	For each source of evidence, present characteristics for which data were charted and provide the citations.	Click here to enter text.
Critical appraisal within sources of evidence	16	If done, present data on critical appraisal of included sources of evidence (see item 12).	Click here to enter text.
Results of individual sources of evidence	17	For each included source of evidence, present the relevant data that were charted that relate to the review questions and objectives.	Click here to enter text.
Synthesis of results	18	Summarize and/or present the charting results as they relate to the review questions and objectives.	Click here to enter text.
**DISCUSSION**			
Summary of evidence	19	Summarize the main results (including an overview of concepts, themes, and types of evidence available), link to the review questions and objectives, and consider the relevance to key groups.	Click here to enter text.
Limitations	20	Discuss the limitations of the scoping review process.	Click here to enter text.
Conclusions	21	Provide a general interpretation of the results with respect to the review questions and objectives, as well as potential implications and/or next steps.	Click here to enter text.
**FUNDING**			
Funding	22	Describe sources of funding for the included sources of evidence, as well as sources of funding for the scoping review. Describe the role of the funders of the scoping review.	Click here to enter text.

JBI = Joanna Briggs Institute; PRISMA-ScR = Preferred Reporting Items for Systematic reviews and Meta-Analyses extension for Scoping Reviews.

* Where *sources of evidence* (see second footnote) are compiled from, such as bibliographic databases, social media platforms, and Web sites.

† A more inclusive/heterogeneous term used to account for the different types of evidence or data sources (e.g., quantitative and/or qualitative research, expert opinion, and policy documents) that may be eligible in a scoping review as opposed to only studies. This is not to be confused with *information sources* (see first footnote).

‡ The frameworks by Arksey and O'Malley (6) and Levac and colleagues (7) and the JBI guidance (4, 5) refer to the process of data extraction in a scoping review as data charting.

§ The process of systematically examining research evidence to assess its validity, results, and relevance before using it to inform a decision. This term is used for items 12 and 19 instead of "risk of bias" (which is more applicable to systematic reviews of interventions) to include and acknowledge the various sources of evidence that may be used in a scoping review (e.g., quantitative and/or qualitative research, expert opinion, and policy document).

From: Tricco AC, Lillie E, Zarin W, O'Brien KK, Colquhoun H, Levac D, et al. PRISMA Extension for Scoping Reviews (PRISMAScR): Checklist and Explanation. Ann Intern Med. 2018;169:467–473. doi: 10.7326/M18-0850.

For peer review only

Supplementary file 2: PRISMA 2009 Flow Diagram (adapted for a scoping review)

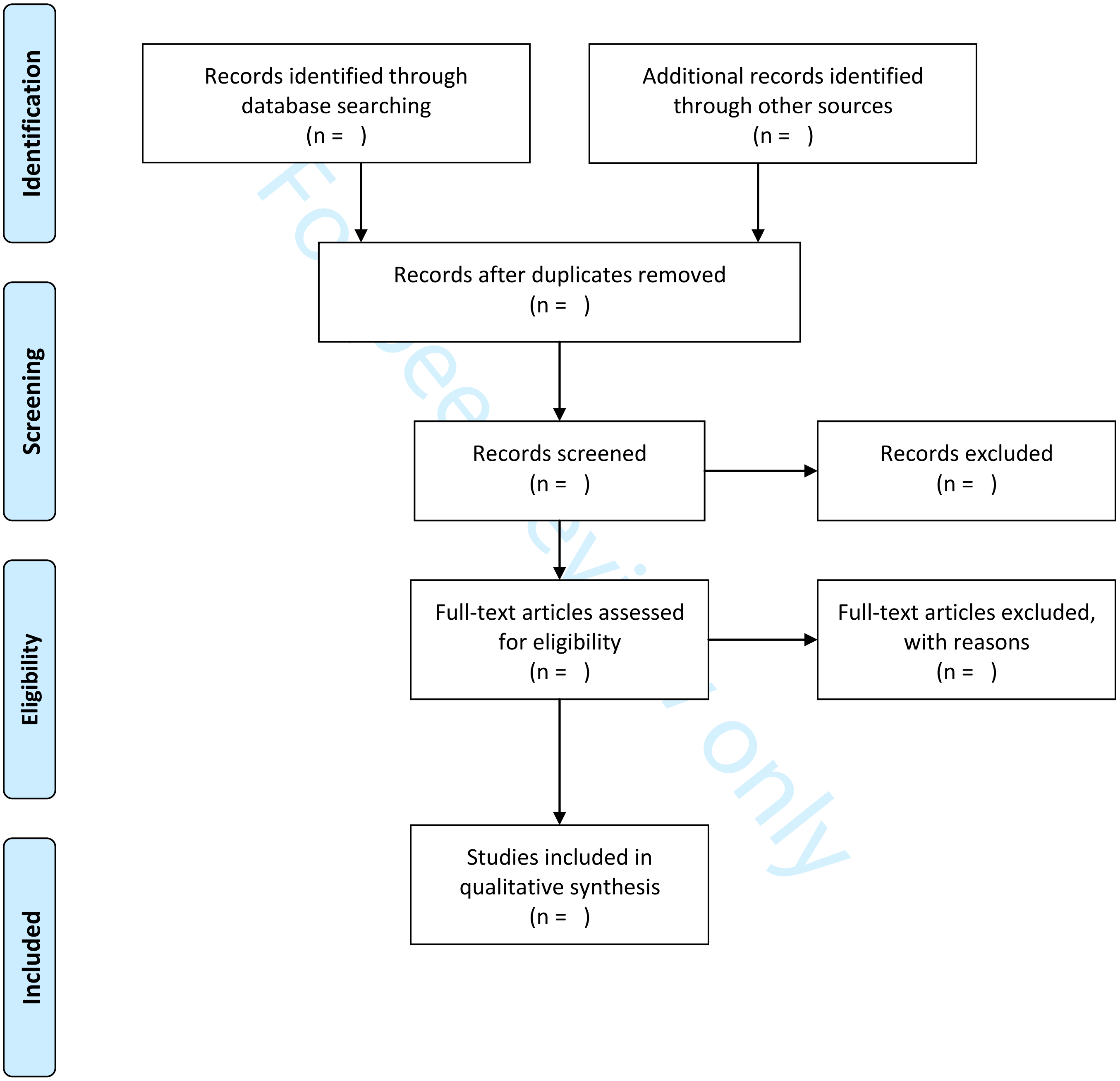

From: Moher D, Liberati A, Tetzlaff J, Altman DG, The PRISMA Group (2009). *P*referred *R*eporting *I*tems for *S*ystematic Reviews and *M*eta-*A*nalyses: The PRISMA Statement. PLoS Med 6(7): e1000097. doi:10.1371/journal.pmed1000097

For more information, visit www.prisma-statement.org.

For peer review only - http://bmjopen.bmj.com/site/about/guidelines.xhtml

Supplementary file 3

Search strategy to be conducted in CINAHL

(((MH "Midwifery+") OR (MH "Students, Nurse Midwifery") OR (MH "Students, Midwifery") OR (MH "Lay Midwifery") OR (MH "Research, Midwifery") OR (MH "Nurse-Midwifery Service") OR (MH "Nurse Midwifery") OR (MH "Midwifery Service+")) AND ((MH "Professional Knowledge+") OR (MH "Clinical Competence+") OR (MH "Professional Competence+") OR (MH "Competency Assessment") OR (MH "Competency Assessment") OR (MH "Education, Competency-Based") OR (MH "Teaching Methods+/EV") OR (MH "Critical Thinking/EV")) AND (impact OR evaluation OR tools OR measuring OR exam* OR test* OR impact*)) OR (MH "Education, Midwifery" AND (evaluation OR impact OR tools OR testing OR exam* OR competen* OR "critical thinking"))

For peer review only

For peer review only - http://bmjopen.bmj.com/site/about/guidelines.xhtml

Supplementary file 4

Data extraction form

Ref. ID	First Author	Year of Publication	Year(s) of Study Coverage	Study Design	Country	Study location	Study setting	Education area of focus	Cadres trained	On-site	Off-site	Online	Lectures	Simulation	Mentorship	Training Facilitator	Identifiable training materials	

For peer review only

For peer review only - http://bmjopen.bmj.com/site/about/guidelines.xhtml

Supplementary file 5
Tools to map education and training material content to ICM Competencies for Midwifery Practice and Lancet Series Quality Maternal and Newborn Care framework

Data collection tool: Mapping to ICM Essential Competencies for Midwifery Practice

**Reference ID**									
**First author**									
**Year of publication**									
**Identified training resource**									
**Category 1: General Competencies**									
**1a: Assume responsibility for own decisions and actions as an autonomous practitioner**									
Knowledge	Principles of accountability and transparency								
	Principles and concepts of autonomy								
	Principles of self-assessment and reflective practice								
	Personal beliefs and their influence on practice								
	Knowledge of evidence-based practices								
Skills and Behaviours	Demonstrate behaviour that upholds the public trust in the profession								
	Participate in self-evaluation, peer review and other quality improvement activities								
	Balance the responsibility of the midwife to provide best care with the autonomy of the woman to make her own decisions								
	Explain the midwife's role in providing care that is based on relevant laws, ethics and evidence.								
**1b: Assume responsibility for self-care and self-development as a midwife**									
Knowledge	Strategies for managing personal safety particularly within the facility or community setting								
Skills and behaviours	Display skills in management of self in relation to time management, uncertainty, change and coping with stress								
	Assume responsibility for personal safety in various practice settings								
	Maintain up-to-date skills and knowledge concerning protocols, guidelines and safe practice								
	Remain current in practice by participating in continuing professional education for example, participating in learning opportunities that apply evidence to practice to improve care such as mortality reviews or policy reviews)								
	Identify and address limitations in personal skill, knowledge, or experience								
	Promote the profession of midwifery, including participation in professional organizations at the local and national level								
**1c: Appropriately delegate aspects of care and provide supervision**									
Knowledge	Policies and regulation related to delegation								
	Supportive strategies to supervise others								
	Role of midwives as preceptors, mentors, supervisors, and role models								
Skills and behaviours	Provide supervision to ensure that practice is aligned with evidence-based clinical practice guidelines								
	Support the profession's growth through participation in midwifery education in the roles of clinical preceptor, mentor, and role model								
**1d: Use research to inform practice**									
Knowledge	Principles of research and evidence-based practice								
	Epidemiologic concepts relevant to maternal and infant health								
	Global recommendations for practice and their evidence base (e.g. World Health Organization guidelines)								
Skills and behaviours	Discuss research findings with women and colleagues								

For peer review only - http://bmjopen.bmj.com/site/about/guidelines.xhtml

Supplementary file 5
Tools to map education and training material content to ICM Competencies for Midwifery Practice and Lancet Series Quality Maternal and Newborn Care framework

	Support research in midwifery by participating in the conduct of research								
**1e: Uphold fundamental human rights of individuals when providing midwifery care**									
Knowledge	Laws and/or codes that protect human rights								
	Sexual, reproductive health rights of women and girls								
	Development of gender identity and sexual orientation								
	Principles of ethics and Human Rights within midwifery practice								
Skills and behaviours	Provide information to women about their sexual and reproductive health rights								
	Inform women about the scope of midwifery practice and women's rights and responsibilities								
	Provide information and support to individuals in complex situations where there are competing ethical principles and rights								
	Practice in accordance with philosophy and code of ethics of the ICM and national standards for health professionals								
	Provide gender sensitive care								
**1f: Adhere to jurisdictional laws, regulatory requirements, and codes of conduct for midwifery practice.**									
Knowledge	The laws and regulations of the jurisdiction regarding midwifery								
	National/state/local community standards of midwifery practice								
	Ethical principles								
	ICM and other midwifery philosophies, values, codes of ethics								
Skills and behaviours	Practise according to legal requirements and ethical principles								
	Meet requirements for maintenance of midwifery registration								
	Protect confidentiality of oral information and written records about care of women and infants								
	Maintain records of care in the manner required by the health authority								
	Comply with all local reporting regulations for birth and death registration								
	Recognize violations of laws, regulations, and ethical codes and take appropriate action								
	Report and document incidents and adverse outcomes as required while providing care								
**1g: Facilitate women to make individual choices about care**									
Knowledge	Cultural norms and practices surrounding sexuality, sexual practices, marriage, the childbearing continuum, and parenting								
	Principles of empowerment								
	Methods of conveying health information to individuals, groups, communities								
Skills and behaviours	Advocate for and support women to be the central decision makers in their care								
	Assist women to identify their needs, knowledge, skills, feelings, and preferences throughout the course of care								
	Provide information and anticipatory guidance about sexual and reproductive health to assist women's decision making								
	Collaborate with women in developing a comprehensive plan of care that respects her preferences and decisions								
**1h: Demonstrate effective interpersonal communication with women and families, health care teams, and community groups**									
Knowledge	Role and responsibilities of midwives and other maternal – infant health providers								
	Principles of effective communication								
	Principles of effectively working in health care teams								
	Cultural practices and beliefs related to childbearing and reproductive health								
	Principles of communication in crisis situations, e.g. grief and loss, emergencies								
Skills and behaviours	Listen to others in an unbiased and empathetic manner								

For peer review only

For peer review only - http://bmjopen.bmj.com/site/about/guidelines.xhtml

Supplementary file 5

Tools to map education and training material content to ICM Competencies for Midwifery Practice and Lancet Series Quality Maternal and Newborn Care framework

	Respect one others' point of view								
	Promote the expression of diverse opinions and perspectives								
	Use the preferred language of the woman or an interpreter to maximise communication								
	Establish ethical and culturally-appropriate boundaries between professional and non-professional relationships								
	Demonstrate cultural sensitivity to women, families, and communities								
	Demonstrate sensitivity and empathy for bereaved women and family members								
	Facilitate teamwork and inter-professional care with other care providers (including students) and community groups/ agencies								
	Establish and maintain collaborative relationships with individuals, agencies, institutions that are part of referral networks								
	Convey information accurately and clearly and respond to the needs of individuals								
**1i: Facilitate normal birth processes in institutional and community settings, including women's homes**									
Knowledge	Normal biologic, psychologic, social, and cultural aspects of reproduction and early life								
	Practices that facilitate and those that interfere with normal processes								
	Policies and protocols about care of women in institutional and community settings								
	Availability of resources in various settings								
	Community views about and utilization of health care facilities and place(s) of birth								
Skills and behaviours	Promote policies and a work culture that values normal birth processes								
	Utilize human and clinical care resources to provide personalized care for women and their infants								
	Provide continuity of care by midwives known to woman								
**1j: Assess the health status, screen for health risks, and promote general health and well-being of women and infants**									
Knowledge	Health needs of women related to reproduction								
	Health conditions that pose risks during reproduction								
	Health needs of infants and common risks								
Skills and behaviours	Conduct a comprehensive assessment of sexual and reproductive health needs								
	Assess risk factors and at-risk behaviour								
	Order, perform, and interpret laboratory and/ or imaging screening tests								
	Exhibit critical thinking and clinical reasoning informed by evidence when promoting health and well being								
	Provide health information and advice tailored to individual circumstances of women and their families								
	Collaborate with women to develop and implement a plan of care								
**1k: Prevent and treat common health problem related to reproduction and early life**									
Knowledge	Common health problems related to sexuality and reproduction								
	Common health problems and deviations from normal of newborn infants								
	Treatment of common health problems								
	Strategies to prevent and control the acquisition and transmission of environmental and communicable diseases								
Skills and behaviours	Maintain/promote safe and hygienic conditions for women and infants								
	Use universal precautions consistently								
	Provide options to women for coping with and treating common health problems								
	Use technology and interventions appropriately to promote health and prevent secondary complications								
	Recognize when consultation or referral is indicated for managing identified health problems, including consultation with other midwives								

For peer review only

For peer review only - http://bmjopen.bmj.com/site/about/guidelines.xhtml

Supplementary file 5

Tools to map education and training material content to ICM Competencies for Midwifery Practice and Lancet Series Quality Maternal and Newborn Care framework

	Include woman in decision-making about referral to other providers and services								
**1l: Recognise abnormalities and complications and institute appropriate treatment and referral**									
Knowledge	Complications/pathologic conditions related to health status								
	Emergency interventions/life-saving therapies								
	Limits of midwifery scope of practice and own experience								
	Available referral systems to access medical and other personnel to manage complications								
	Community/facility plans and protocols for accessing resources in timely manner								
Skills and behaviours	Maintain up-to-date knowledge, life-saving skills, and equipment for responding to emergency situations								
	Recognize situations requiring expertise beyond midwifery care								
	Maintain communication with women about nature of problem, actions taken, and referral if indicated								
	Determine the need for immediate intervention and respond appropriately								
	Implement timely and appropriate intervention, inter-professional consultation and/or timely referral taking account of local circumstances								
	Provide accurate oral and written information to other care providers when referral is made								
	Collaborate with decision-making if possible and appropriate								
**1m: Care for women who experience physical and sexual violence and abuse**									
Knowledge	Socio-cultural, behavioural, and economic conditions that often accompany violence and abuse								
	Resources in community to assist women and children								
	Risks of disclosure								
Skills and behaviours	Protect privacy and confidentiality								
	Provide information to all women about sources of help regardless of whether there is disclosure about violence								
	Inquire routinely about safety at home, at work								
	Recognize potential signs of abuse from physical appearance, emotional affect, related risk behaviours such as substance abuse								
	Provide special support for adolescents and victims of gender-based violence including rape								
	Refer to community resources, assist in locating safe setting as needed								
**Category 3: Care during labour and birth**									
**3a: Promote physiological labour and birth**									
Knowledge	Anatomy of maternal pelvis and fetus; mechanisms of labour for different fetal presentations								
	Physiologic onset and progression of labour								
	Evidence informed intrapartum care policies and guidelines, including avoidance of routine interventions in normal labour and birth								
	Cultural and social beliefs and traditions about birth								
	Signs and behaviours of labour progress; factors that impede labour progress								
	Methods of assessing fetus during labour								
Skills and behaviours	Provide care for a woman in the birth setting of her choice, following policies and protocols								
	Obtain relevant obstetric and medical history								
	Perform and interpret focused physical examination of the woman and fetus								
	Order and interpret laboratory tests if needed								
	Assess woman's physical and behavioural responses to labour								
	Provide information, support, and encouragement to woman and support persons throughout labour and birth								

For peer review only - http://bmjopen.bmj.com/site/about/guidelines.xhtml

Supplementary file 5

Tools to map education and training material content to ICM Competencies for Midwifery Practice and Lancet Series Quality Maternal and Newborn Care framework

	Provide respectful one-to-one care								
	Encourage freedom of movement and upright positions								
	Provide nourishment and fluids								
	Offer and support woman to use strategies for coping with labour pain, e.g. controlled breathing, water immersion, relaxation, massage, and pharmacologic modalities when needed								
	Assess regularly parameters of maternal-fetal status, and e.g. vital signs, contractions, cervical changes, and fetal descent								
	Use labour progress graphic display to record findings and assist in detecting complications, e.g. labour delay, fetal compromise, maternal exhaustion, hypertension, infection								
	Augment uterine contractility judiciously using non-pharmacological or pharmacological agents to prevent non-progressive labour								
	Prevent unnecessary routine interventions, e.g. amniotomy, electronic fetal monitoring, directed closed glottis pushing, episiotomy								
**3b: Manage a safe spontaneous vaginal birth; prevent, detect and stabilize complications**									
Knowledge	Manage a safe spontaneous vaginal birth								
	Evidence about conduct of third stage, including use of uterotonics								
	Potential complications and their immediate treatment e.g. shoulder dystocia,and excessive bleeding, fetal compromise, eclampsia, retained placenta								
	Management of emergencies as covered in emergency skills trainingprogrammes such as BEmONC,HMS								
	Signs of placental separation; appearance of normal placenta, membranes,and umbilical cord								
	Types of perineal and vaginal trauma requiring repair and suturing techniques								
Skills and behaviours	Support the woman to give birth in her position of choice								
	Ensure clean environment, presence of clean necessary supplies and sourceof warmth								
	Coach woman about pushing to control expulsion of presenting part, avoid routine episiotomy								
	Undertake appropriate manoeuvers and use maternal position to facilitatevertex, face, or breech birth								
	Expedite birth in presence of fetal distress								
	Delay cord clamping								
	Manage nuchal cord								
	Assess immediate condition of newborn								
	Provide skin to skin contact and warm environment								
	Deliver placenta and membranes and inspect for completeness								
	Assess uterine tone, maintain firm contraction, and estimate and record maternal blood loss; manage excessive blood loss including administration of uterotonics								
	Inspect vaginal and perineal areas for trauma, and repair as needed,following policies and protocols								
	Provide first line measures to treat or stabilise identified conditions								
	Refer for continuing treatment of any complications as needed								
**3c: Provide care of the newborn immediately after birth**									
Knowledge	Normal transition to extra-uterine environment								
	Scoring systems to assess newborn status								

For peer review only - http://bmjopen.bmj.com/site/about/guidelines.xhtml

Supplementary file 5

Tools to map education and training material content to ICM Competencies for Midwifery Practice and Lancet Series Quality Maternal and Newborn Care framework

	Signs indicating need for immediate actions to assist transition								
	Interventions to establish breathing and circulation as covered in training programs such as HBS								
	Appearance and behaviour of healthy newborn infant								
	Method of assessing gestational age of newborn infant								
	Needs of small for gestational age and low birth weight infants								
Skills and behaviour	Use standardized method to assess newborn condition in the first minutes of life (Apgar or other); refer if needed								
	Institute actions to establish and support breathing and oxygenation, refer for continuing treatment as needed								
	Provide a safe warm environment for initiating breastfeeding and attachment (bonding) in the first hour of life								
	Conduct a complete physical examination of newborn in presence of mother/family; explain findings and expected changes e.g. colour of extremities, moulding of head. Refer for abnormal findings.								
	Institute newborn prophylaxis e.g. ophthalmic infection, and hemorrhagic disease, according to policies and guidelines								
	Promote care by mother, frequent feeding and close observation								
	Involve partner/support persons in providing newborn care								

For peer review only

For peer review only - http://bmjopen.bmj.com/site/about/guidelines.xhtml

Supplementary file 5

Tools to map education and training material content to ICM Competencies for Midwifery Practice and Lancet Series Quality Maternal and Newborn Care framework

Data collection tool: Mapping to Lancet Series Quality Maternal and Newborn Care framework

**Reference ID**									
**First author**									
**Year of publication**									
**Identified training resource**									
**Practice Categories**									
For all childbearing women and infants	Education								
	Information								
	Health Promotion								
	Assessment								
	Screening								
	Care planning								
	Promotion of normal processes, prevention of complications								
For childbearing women and infants with complications	First line management of complications								
	Medical obstetric neonatal services								
**Organisation of care**									
For all childbearing women and infants AND for all childbearing women and infants with complications	Available								
	Accessible								
	Acceptable								
	Good quality services								
	Adequate resources								
	Competent workforce								
	Continuity								
	Services integrated across community and facilities								
**Values**									
For all childbearing women and infants AND for all childbearing women and infants with complications	Respect								
	Communication								
	Community knowledge								
	Understanding								
	Care tailored to women's circumstances and needs								
**Philosophy**									
For all childbearing women and infants AND for all childbearing women and infants with complications	Optimising biological, psychological, social and cultural processes								
	Strengthening women's capabilities								
	Expectant management, using interventions only when indicated								
**Care providers**									
For all childbearing women and infants AND for all childbearing women and infants with complications	Practitioners who combine clinical knowledge and skills with interpersonal and cultural competence								
	Division of roles and responsibility based on need, competencies and resources								

For peer review only - http://bmjopen.bmj.com/site/about/guidelines.xhtml

BMJ Open

Protocol for a scoping review to identify and map in-service education and training materials for midwifery care in sub-Saharan Africa from 2000-2020

Journal:	*BMJ Open*
Manuscript ID	bmjopen-2020-047118.R1
Article Type:	Protocol
Date Submitted by the Author:	13-Feb-2021
Complete List of Authors:	Welsh, Joanne; Hannover Medical School, Midwifery Research and Education Unit<br>Gross, MM; Hannover Medical School, Midwifery Research and Education Unit<br>Hanson, Claudia; Karolinska Institute, Dept Public Global Health<br>Hounkpatin , Hashim; Centre de Recherche en Reproduction Humaine et en Démographie (CERRHUD),<br>Moller, Ann-Beth; University of Gothenburg Institute of Medicine,
**Primary Subject Heading**:	Public health
Secondary Subject Heading:	Obstetrics and gynaecology
Keywords:	OBSTETRICS, PUBLIC HEALTH, MEDICAL EDUCATION & TRAINING

SCHOLARONE™
Manuscripts

I, the Submitting Author has the right to grant and does grant on behalf of all authors of the Work (as defined in the below author licence), an exclusive licence and/or a non-exclusive licence for contributions from authors who are: i) UK Crown employees; ii) where BMJ has agreed a CC-BY licence shall apply, and/or iii) in accordance with the terms applicable for US Federal Government officers or employees acting as part of their official duties; on a worldwide, perpetual, irrevocable, royalty-free basis to BMJ Publishing Group Ltd ("BMJ") its licensees and where the relevant Journal is co-owned by BMJ to the co-owners of the Journal, to publish the Work in this journal and any other BMJ products and to exploit all rights, as set out in our licence.

The Submitting Author accepts and understands that any supply made under these terms is made by BMJ to the Submitting Author unless you are acting as an employee on behalf of your employer or a postgraduate student of an affiliated institution which is paying any applicable article publishing charge ("APC") for Open Access articles. Where the Submitting Author wishes to make the Work available on an Open Access basis (and intends to pay the relevant APC), the terms of reuse of such Open Access shall be governed by a Creative Commons licence – details of these licences and which Creative Commons licence will apply to this Work are set out in our licence referred to above.

Other than as permitted in any relevant BMJ Author's Self Archiving Policies, I confirm this Work has not been accepted for publication elsewhere, is not being considered for publication elsewhere and does not duplicate material already published. I confirm all authors consent to publication of this Work and authorise the granting of this licence.

For peer review only

Protocol for a scoping review to identify and map in-service education and training materials for midwifery care in sub-Saharan Africa from 2000-2020

Author names

Joanne Welsh[1], Mechthild M. Gross[1], Claudia Hanson[2,3], Hashim Hounkpatin[4], Ann-Beth Moller[5].

1. Midwifery Research and Education Unit, Hannover Medical School. Hannover, Germany.

2. Global Public Health, Karolinska Institute. Stockholm, Sweden.

3. Department of Disease Control. London School of Hygiene and Tropical Medicine. London, United Kingdom.

4. Centre de Recherche en Reproduction Humaine et en Démographie (CERRHUD). Cotonou, Benin.

5. School of Public Health and Community Medicine, Institute of Medicine, University of Gothenburg, Gothenburg, Sweden.

Corresponding author: Ann-Beth Moller

School of Public Health and Community Medicine, Institute of Medicine, University of Gothenburg. Box 100, SE-405 30, Gothenburg, Sweden.

Email: ann-beth.moller.2@gu.se

Word Count: 1849 words

For peer review only - http://bmjopen.bmj.com/site/about/guidelines.xhtml

Abstract

Introduction

Maternal and neonatal mortality are disproportionally high in low- and middle-income countries. In 2017 the global maternal mortality ratio (MMR) was estimated to be 211 per 100,000 live births. An estimated 66% of these deaths occurred in sub-Saharan Africa. Training programmes that aim to prepare providers of midwifery care vary considerably across sub-Saharan Africa in terms of length, content and quality. To overcome the shortfalls of pre-service training and support the provision of quality care, in-service training packages for providers of midwifery care have been developed and implemented in many countries in sub-Saharan Africa. We aim to identify what in-service education and training materials have been used for providers of midwifery care between 2000-2020 and map their content to the International Confederation of Midwives' Essential Competencies for Midwifery Practice (ICM Competencies), and the Lancet Midwifery Series Quality Maternal and Newborn Care (QMNC) Framework.

Methods and analysis

A search will be conducted for the years 2000-2020 in Cumulative Index of Nursing and Allied Health Literature (CINAHL), PubMed/MEDLINE, Social Science Citation Index, African Index Medicus and Google Scholar. A manual search of reference lists from identified studies and a hand search of literature from international partner organisations will be performed. Information retrieved will include study context, providers trained, focus of training, and design of training. Original content of identified education and training materials will be obtained and mapped to the ICM Competencies and the Lancet Series QMNC.

For peer review only - http://bmjopen.bmj.com/site/about/guidelines.xhtml

Ethics and dissemination

A scoping review is a secondary analysis of published literature and does not require ethical approval. This scoping review will give an overview of the education and training materials used for in-service training for providers of midwifery care in sub-Saharan Africa. Mapping the content of these education and training materials to the ICM Competencies and The Lancet Series QMNC will allow us to assess their appropriateness. Findings from the review will be reflected to stakeholders involved in the design and implementation of such materials. Additionally, findings will be published in a peer-reviewed journal, and used to inform the design and content of an in-service training package for providers of midwifery care as part of the Action Leveraging Evidence to Reduce perinatal morTality and morbidity (ALERT) study, (https://alert.ki.se/) a multi country study in Benin, Malawi, Tanzania and Uganda.

Strengths and limitations of this study

- This scoping review will give a comprehensive overview of the education and training materials used for in-service training for providers of midwifery care in sub-Saharan Africa.
- To our knowledge this is the first review that aims to map the content of in-service education and training materials used for providers of midwifery care in sub-Saharan Africa to the ICM Essential Competencies and The Lancet Series Quality Maternal and Newborn Care Framework.
- This scoping review may miss in-service training materials that have not been reported in journals or published in the grey literature.

INTRODUCTION

A transformative agenda for maternal and newborn health has been laid out as part of the Sustainable Development Goals (SDGs); “to reduce the global maternal mortality ratio (MMR) to less than 70 per 100 000 live births by 2030” (SDG 3.1.) and “end preventable deaths of newborns, with all countries aiming

For peer review only - http://bmjopen.bmj.com/site/about/guidelines.xhtml

to reduce neonatal mortality to at least as low as 12 per 1000 live births" (SDG 3.2).[1] Maternal and neonatal mortality are disproportionally high in low- and middle-income countries (LMICs). In 2017 the global maternal mortality ratio (MMR) was estimated to be 211 per 100,000 live births. An estimated 66% of these deaths occurred in sub-Saharan Africa, whose regional MMR in 2017 was 542.[2] Similarly, despite a global neonatal mortality rate of 17 per 1,000 live births in 2019 this rate in sub-Saharan Africa was 27 per 1,000.[3] With access to evidence based, quality care the majority of these deaths are preventable. Within an enabling environment, midwifery care is effective in improving care and supports the international agenda to end preventable maternal and newborn deaths. [4 5] While today many more women give birth with a skilled birth attendant,[6] mortality reductions have been less than anticipated.[7] This paradox might be explained in relation to the complexity of provision of high-quality maternity care which is reliant on several factors including an appropriately trained and qualified health workforce who are equitably distributed, an enabling environment which includes access to essential equipment, medications and supplies as well as social and cultural aspects of care.

The workforce providing midwifery care is diverse. A study by Hobbs et al.[8] identified 102 unique cadre names from 36 LMICs who were involved in the provision of pregnancy, childbirth and postpartum care. For this review the term "provider of midwifery care" is used. See box 1. for the definition of provider of midwifery care used in this review.

<u>Definition of provider of midwifery care</u>

Providers of midwifery care are competent maternal and newborn health professionals educated, trained and regulated to national and/or international standards. They provide skilled, evidence-based and compassionate care to women, newborns and families.

Providers of midwifery care:

- Promote and facilitate normal physiological, social and cultural processes throughout the childbearing continuum with a continuity of care philosophy;
- Seek to prevent and manage maternal and newborn complications;
- Consult and refer to other health services when required; and
- Respect women's individual circumstances and views, providing sensitive and dignified care.

For peer review only

Box 1: Definition of midwifery care [9, 10]

Substantial heterogeneity exists in the length, content and quality of training that different providers of midwifery care receive to prepare them for the workforce, as well as in their ability to perform obstetric and neonatal functions. [8, 11] Inadequate training has been found to diminish the competence and confidence of providers of midwifery care.[12] In-service education therefore plays an important role in filling gaps that arise from inadequate pre-service education. It also acts to support continuous professional development for providers of midwifery care. A number of in-service training packages for providers of midwifery care have been developed and implemented in sub-Saharan Africa as a means to support the provision of quality maternity care.[13-16] Many in-service training packages focus on emergency obstetric care,[13-17] with few focusing on normal childbirth,[18] and dignified and respectful care.[19]

It is imperative that in-service education is evidence based, of high quality and promotes both autonomy and multidisciplinary team working. The International Confederation of Midwives[20] "Essential Competencies for Midwifery Practice" outline the minimum set of knowledge, skills and professional behaviour expected of an individual completing their midwifery training and joining the workforce. These essential competencies are helpful in the design and evaluation of ongoing in-service training for providers of midwifery care. Additionally, the Lancet Series Quality Maternal and Newborn Care (QMNC) framework describes the components of care that are critical to the provision of best quality maternity care and places women's views and experiences at its core.[9] The framework identifies effective practices, the organization of care, the philosophy and values of care providers and the characteristics of care providers as essential components to the provision of quality care. The framework is designed to be relevant to any setting and as such has been recommended as a structure around which improvements in midwifery can be made globally.[21]

For peer review only - http://bmjopen.bmj.com/site/about/guidelines.xhtml

This review is a component of the Action Leveraging Evidence to Reduce perinatal morTality and morbidity in sub-Saharan Africa (ALERT) project (Trial registration number: PACTR202006793783148).[22] The objective of the ALERT project is to develop and evaluate a multifaceted intervention to i) strengthen the implementation of evidence-based interventions and responsive care and, ii) reduce in-facility perinatal mortality and morbidity through a multidisciplinary approach in Benin, Malawi, Tanzania and Uganda. To achieve this, the ALERT project aims to develop and implement a co-designed in-service midwifery training package with a focus on basic intrapartum care. To inform this process this review aims to identify and map in-service education and training materials for providers of midwifery care that are available and have been used in sub-Saharan Africa since 2000.

METHODS AND ANALYSIS

Patient and Public Involvement

No patient involved

Study design

Due to the nature of the research question a scoping review design was chosen to allow for identification and mapping of existing in-service education and training materials. This scoping review follows the steps as outlined by Arksey and O'Malley.[23] The Preferred Reporting Items for Systematic reviews and Meta-Analyses extension for Scoping Reviews (PRISMA-ScR) Checklist (see supplementary file 1) will be used to present the findings of the scoping review, and the PRISMA flow diagram (see supplementary file 2) will be used to map out the phases of literature identification; the number of records identified, and those included and excluded with reasons given for exclusions.[24]

Research questions

For peer review only - http://bmjopen.bmj.com/site/about/guidelines.xhtml

The objectives of our study are to identify, and map in-service education and training materials designed for providers of midwifery care in sub-Saharan Africa from 2000-2020. The scoping review aims to address the following questions:

1. What in-service education and training materials are available and have been used for providers of midwifery care in sub-Saharan Africa from 2000-2020?
2. How does the content of these in-service education and training materials align with the International Confederation of Midwives Essential Competencies for Midwifery Practice as well as in relation to the Lancet Midwifery Series QMNC Framework?

Inclusion criteria

The research questions will be assessed, and studies will be selected specific to the following Population, Concept, Study Design and Context criteria presented in Table 1. All quantitative studies and grey literature that include information on available in-service education and training materials used for providers of maternity care in sub-Saharan Africa will be included in the review. Evidence in the field of clinical care changes frequently and new guidelines and recommendations are updated when new knowledge becomes available. Some guidelines are even "living" guidelines as evidence is constantly emerging (e.g. clinical care in relation to COVID-19). As the focus of the scoping review is on training materials based on evidence, only those developed and used after 2000 onwards will be included.

Table 1. Inclusion criteria for identifying eligible studies

	**Inclusion Criteria**
**Population**	Any in-service training on midwifery care aimed at any health professional who provides midwifery care.
**Concept**	Mapping the content of in-service training materials used for providers of midwifery care to the ICM Essential Competencies and The Lancet Series QMNC Framework.
**Study Design**	Quantitative studies and reports/documents that report on the implementation of in-service training materials and in-service training resources identified through searches of grey literature.

For peer review only - http://bmjopen.bmj.com/site/about/guidelines.xhtml

Context	In-service education in sub-Saharan Africa.

Exclusion criteria

Articles will not be eligible for inclusion in the scoping review if:

1. The country in which the in-service education and training has taken place is not located within sub-Saharan Africa.
2. The health personnel who have undertaken the in-service education and training is not considered to be a provider of midwifery care.
3. The content of the in-service education and training does not relate to the provision of midwifery care.
4. The in-service education and training materials were created before 2000
5. There are no details provided on the content of the education and training sessions.

Search strategy

The search strategy will be conducted for all relevant existing literature, without language restrictions, based on search terms relating to the research questions and restricted to the years 2000-2020 using the following online bibliographic databases: Cumulative Index of Nursing and Allied Health Literature (CINAHL), PubMed/MEDLINE, Social Science Citation Index, African Index Medicus, and Google Scholar. Grey literature searches will be performed and include organizations known to be active in global health improvement (i.e. United Nations Population Fund [UNFPA], World Health Organization [WHO], Johns Hopkins Program for International Education in Gynecology and Obstetrics [JHPIEGO], International Confederation of Midwives [ICM], International Federation of Gynecology and Obstetrics [FIGO], International Pediatric Association [IPA]). The reference list of all eligible studies will be hand-searched to

For peer review only - http://bmjopen.bmj.com/site/about/guidelines.xhtml

identify any additional relevant studies. Examples of the preliminary search terms and strategy to be conducted in CINAHL are outlined in supplementary file 3.

Study selection

Following the aforementioned comprehensive search strategy, article titles and abstracts will be screened and eligibility for inclusion assessed independently by two reviewers (ABM and JW). Any disagreements will be resolved by a third reviewer. Screened abstracts identified for inclusion will have their full texts independently screened by ABM and JW. As the scoping review aims to map the content of in-service training materials we will not assess the quality of the studies reporting on the in-service training. All studies meeting inclusion criteria will therefore be included in the review. Full text articles that are excluded at the screening stage will have reasons for exclusion documented. The Covidence[25] software will be used for both title/abstract and full text screening.

Data extraction and analysis

A data collection tool will be used to extract required information (see supplementary file 4). Information retrieved will include study context (country and health care setting). Details of the cadres of providers of midwifery care trained will be recorded. Further data will be collected regarding the focus of the training and design of the training (formal education sessions, training facilitators, on-site training, off-site training, use of simulation, ongoing mentorship, online training). The data extraction form will contain additional fields to allow for flexibility should any other categories of training design arise that the authors had not initially considered.

Education and training materials identified in the scoping review will be sought by the reviewers. If the reviewers are unable to access the materials, they will contact the authors and ask them to share

For peer review only - http://bmjopen.bmj.com/site/about/guidelines.xhtml

materials. This will allow original content to be mapped to the ICM Essential Competencies for Midwifery Practice[20] and the Lancet Series QMNC[9]. The tools used to map content of education and training materials can be found in supplementary file 5. Descriptive statistics will be used to summarise the findings on how the identified in-service training materials contain content pertaining to the ICM Essential Competencies and the Lancet Series QMNC framework.

DISSEMINATION AND ETHICS

This scoping review will give a comprehensive overview of the education and training materials used for in-service training of providers of midwifery care in sub-Saharan Africa. To our knowledge this is the first review that aims to map the content of education and training materials used for providers of midwifery care in sub-Saharan Africa to the ICM Essential Competencies and The Lancet Series QMNC framework. To ensure future in-service education and training materials are of quality and designed to adhere to the ICM Essential Competencies and the Lancet Series QMNC framework, findings from the scoping review will be reflected to stakeholders involved in the design and implementation of such materials and will be used to inform the development of the ALERT in-service midwifery training package. The authors aim to disseminate findings from the scoping review by publication in a peer-reviewed journal. Scoping reviews collect and examine data from existing literature and therefore do not require prior ethical approval.

Author's contributions

JW wrote the original draft. ABM contributed to the conception of the study and substantively revised the manuscript. JW and ABM developed the data extraction form. All authors JW, ABM, MG, CH and HH have contributed to the manuscript and read and approved the final version.

Funding statement

For peer review only - http://bmjopen.bmj.com/site/about/guidelines.xhtml

This study is part of the ALERT-project which is funded by the European Commission's Horizon 2020 (No 847824) under a call for Implementation research for maternal and child health. The contents of this article are solely the responsibility of the authors and do not reflect the views of the EU.

Competing Interests

The authors declare that they have no competing interests.

Supplementary files

Supplementary file 1: The Preferred Reporting Items for Systematic reviews and Meta-Analyses extension for Scoping Reviews (PRISMA-ScR) Checklist.

Supplementary file 2: The Preferred Reporting Items for Systematic reviews and Meta-Analyses (PRISMA) flow diagram.

Supplementary file 3: Preliminary search terms and strategy to be conducted in CINAHL.

Supplementary file 4: Data extraction form.

Supplementary file 5: Tools to map education and training material content to ICM Competencies for Midwifery Practice and Lancet Series Quality Maternal and Newborn Care framework.

References

1. Transforming our world: the 2030 Agenda for Sustainable Development. Resolution adopted by the General Assembly on 25 September 2015. New York (NY): United Nations, 2015.
2. WHO, UNICEF, UNFPA, et al. Trends in maternal mortality: 2000 to 2017: estimates by WHO, UNICEF, UNFPA, The World Bank Group and the United Nations Population Division. Geneva: World Health Organization, 2019.
3. United Nations Inter-agency Group for Child Mortality Estimation. Levels and Trends in Child Mortality: Report 2020, Estimates developed by the United Nations Inter-agency Group for Child Mortality Estimation. New York, 2020.
4. Homer CS, Friberg IK, Dias MA, et al. The projected effect of scaling up midwifery. *Lancet* 2014;384(9948):1146-57. doi: 10.1016/s0140-6736(14)60790-x [published Online First: 2014/06/27]

For peer review only - http://bmjopen.bmj.com/site/about/guidelines.xhtml

5. Sandall J, Soltani H, Gates S, et al. Midwife-led continuity models versus other models of care for childbearing women. *Cochrane Database Syst Rev* 2016;4:Cd004667. doi: 10.1002/14651858.CD004667.pub5 [published Online First: 2016/04/29]
6. Montagu D, Sudhinaraset M, Diamond-Smith N, et al. Where women go to deliver: understanding the changing landscape of childbirth in Africa and Asia. *Health Policy Plan* 2017;32(8):1146-52. doi: 10.1093/heapol/czx060 [published Online First: 2017/05/26]
7. Ng M, Misra A, Diwan V, et al. An assessment of the impact of the JSY cash transfer program on maternal mortality reduction in Madhya Pradesh, India. *Glob Health Action* 2014;7:24939. doi: 10.3402/gha.v7.24939 [published Online First: 2014/12/06]
8. Hobbs AJ, Moller AB, Kachikis A, et al. Scoping review to identify and map the health personnel considered skilled birth attendants in low-and-middle income countries from 2000-2015. *PLoS One* 2019;14(2):e0211576. doi: 10.1371/journal.pone.0211576 [published Online First: 2019/02/02]
9. Renfrew MJ, McFadden A, Bastos MH, et al. Midwifery and quality care: findings from a new evidence-informed framework for maternal and newborn care. *Lancet* 2014;384(9948):1129-45. doi: 10.1016/s0140-6736(14)60789-3 [published Online First: 2014/06/27]
10. World Health Organization. Defining competent maternal and newborn health professionals. Geneva, 2018.
11. Michel-Schuldt M, McFadden A, Renfrew M, et al. The provision of midwife-led care in low-and middle-income countries: An integrative review. *Midwifery* 2020;84:102659. doi: 10.1016/j.midw.2020.102659 [published Online First: 2020/02/18]
12. Filby A, McConville F, Portela A. What Prevents Quality Midwifery Care? A Systematic Mapping of Barriers in Low and Middle Income Countries from the Provider Perspective. *PLoS One* 2016;11(5):e0153391. doi: 10.1371/journal.pone.0153391 [published Online First: 2016/05/03]
13. Evans CL, Bazant E, Atukunda I, et al. Peer-assisted learning after onsite, low-dose, high-frequency training and practice on simulators to prevent and treat postpartum hemorrhage and neonatal asphyxia: A pragmatic trial in 12 districts in Uganda. *PLoS One* 2018;13(12):e0207909. doi: 10.1371/journal.pone.0207909 [published Online First: 2018/12/18]
14. Egenberg S, Masenga G, Bru LE, et al. Impact of multi-professional, scenario-based training on postpartum hemorrhage in Tanzania: a quasi-experimental, pre- vs. post-intervention study. *BMC Pregnancy Childbirth* 2017;17(1):287. doi: 10.1186/s12884-017-1478-2 [published Online First: 2017/09/07]
15. Ameh CA, Kerr R, Madaj B, et al. Knowledge and Skills of Healthcare Providers in Sub-Saharan Africa and Asia before and after Competency-Based Training in Emergency Obstetric and Early Newborn Care. *PLoS One* 2016;11(12):e0167270. doi: 10.1371/journal.pone.0167270 [published Online First: 2016/12/23]
16. Crofts JF, Mukuli T, Murove BT, et al. Onsite training of doctors, midwives and nurses in obstetric emergencies, Zimbabwe. *Bull World Health Organ* 2015;93(5):347-51. doi: 10.2471/blt.14.145532 [published Online First: 2015/08/01]
17. Gavine A, MacGillivray S, McConville F, et al. Pre-service and in-service education and training for maternal and newborn care providers in low- and middle-income countries: An evidence review and gap analysis. *Midwifery* 2019;78:104-13. doi: 10.1016/j.midw.2019.08.007 [published Online First: 2019/08/17]
18. Makowiecka K, Achadi E, Izati Y, et al. Midwifery provision in two districts in Indonesia: how well are rural areas served? *Health Policy Plan* 2008;23(1):67-75. doi: 10.1093/heapol/czm036 [published Online First: 2007/11/01]
19. Fujita N, Perrin XR, Vodounon JA, et al. Humanised care and a change in practice in a hospital in Benin. *Midwifery* 2012;28(4):481-8. doi: 10.1016/j.midw.2011.07.003 [published Online First: 2011/09/20]

For peer review only

For peer review only - http://bmjopen.bmj.com/site/about/guidelines.xhtml

20. The International Confederation of Midwives. The International Confederation of Midwives (ICM) Essential Competencies for Midwifery Practice Framework 2019.
21. Bharj KK, Luyben A, Avery MD, et al. An agenda for midwifery education: Advancing the state of the world's midwifery. *Midwifery* 2016;33:3-6. doi: 10.1016/j.midw.2016.01.004 [published Online First: 2016/01/27]
22. Action Leveraging Evidence to reduce perinatal Mortality and morbidity in sub-Saharan Africa project (ALERT) Sweden: Karolinska Institute; 2020 [Available from: https://ki.se/en/gph/the-alert-intervention-research-project accessed 13 April 2020.
23. Arksey H OML. Scoping studies: towards a methodological framework. *Int J Soc Res Methodol* 2005(8):19-32.
24. Liberati A, Altman DG, Tetzlaff J, et al. The PRISMA statement for reporting systematic reviews and meta-analyses of studies that evaluate healthcare interventions: explanation and elaboration. *BMJ* 2009;339:b2700. doi: 10.1136/bmj.b2700 [published Online First: 2009/07/23]
25. Covidence 2020 [Available from: https://www.covidence.org/].

For peer review only

For peer review only - http://bmjopen.bmj.com/site/about/guidelines.xhtml

Supplementary file 1: Preferred Reporting Items for Systematic reviews and Meta-Analyses extension for Scoping Reviews (PRISMA-ScR) Checklist

SECTION	ITEM	PRISMA-ScR CHECKLIST ITEM	REPORTED ON PAGE #
**TITLE**			
Title	1	Identify the report as a scoping review.	1
**ABSTRACT**			
Structured summary	2	Provide a structured summary that includes (as applicable): background, objectives, eligibility criteria, sources of evidence, charting methods, results, and conclusions that relate to the review questions and objectives.	2
**INTRODUCTION**			
Rationale	3	Describe the rationale for the review in the context of what is already known. Explain why the review questions/objectives lend themselves to a scoping review approach.	3-6
Objectives	4	Provide an explicit statement of the questions and objectives being addressed with reference to their key elements (e.g., population or participants, concepts, and context) or other relevant key elements used to conceptualize the review questions and/or objectives.	7
**METHODS**			
Protocol and registration	5	Indicate whether a review protocol exists; state if and where it can be accessed (e.g., a Web address); and if available, provide registration information, including the registration number.	NA
Eligibility criteria	6	Specify characteristics of the sources of evidence used as eligibility criteria (e.g., years considered, language, and publication status), and provide a rationale.	7-8
Information sources*	7	Describe all information sources in the search (e.g., databases with dates of coverage and contact with authors to identify additional sources), as well as the date the most recent search was executed.	8-9
Search	8	Present the full electronic search strategy for at least 1 database, including any limits used, such that it could be repeated.	Supplementary file 3
Selection of sources of evidence†	9	State the process for selecting sources of evidence (i.e., screening and eligibility) included in the scoping review.	7-8
Data charting process‡	10	Describe the methods of charting data from the included sources of evidence (e.g., calibrated forms or forms that have been tested by the team before their use, and whether data charting was done independently or in duplicate) and any processes for obtaining and confirming data from investigators.	9
Data items	11	List and define all variables for which data were sought and any assumptions and simplifications made.	NA
Critical appraisal of individual sources of evidence§	12	If done, provide a rationale for conducting a critical appraisal of included sources of evidence; describe the methods used and how this information was used in any data synthesis (if appropriate).	NA
Synthesis of results	13	Describe the methods of handling and summarizing the data that were charted.	NA

St. Michael's
Inspired Care.
Inspiring Science.

For peer review only - http://bmjopen.bmj.com/site/about/guidelines.xhtml

SECTION	ITEM	PRISMA-ScR CHECKLIST ITEM	REPORTED ON PAGE #
**RESULTS**			
Selection of sources of evidence	14	Give numbers of sources of evidence screened, assessed for eligibility, and included in the review, with reasons for exclusions at each stage, ideally using a flow diagram.	NA
Characteristics of sources of evidence	15	For each source of evidence, present characteristics for which data were charted and provide the citations.	NA
Critical appraisal within sources of evidence	16	If done, present data on critical appraisal of included sources of evidence (see item 12).	NA
Results of individual sources of evidence	17	For each included source of evidence, present the relevant data that were charted that relate to the review questions and objectives.	NA
Synthesis of results	18	Summarize and/or present the charting results as they relate to the review questions and objectives.	NA
**DISCUSSION**			
Summary of evidence	19	Summarize the main results (including an overview of concepts, themes, and types of evidence available), link to the review questions and objectives, and consider the relevance to key groups.	NA
Limitations	20	Discuss the limitations of the scoping review process.	NA
Conclusions	21	Provide a general interpretation of the results with respect to the review questions and objectives, as well as potential implications and/or next steps.	NA
**FUNDING**			
Funding	22	Describe sources of funding for the included sources of evidence, as well as sources of funding for the scoping review. Describe the role of the funders of the scoping review.	11

JBI = Joanna Briggs Institute; PRISMA-ScR = Preferred Reporting Items for Systematic reviews and Meta-Analyses extension for Scoping Reviews.
* Where *sources of evidence* (see second footnote) are compiled from, such as bibliographic databases, social media platforms, and Web sites.
† A more inclusive/heterogeneous term used to account for the different types of evidence or data sources (e.g., quantitative and/or qualitative research, expert opinion, and policy documents) that may be eligible in a scoping review as opposed to only studies. This is not to be confused with *information sources* (see first footnote).
‡ The frameworks by Arksey and O'Malley (6) and Levac and colleagues (7) and the JBI guidance (4, 5) refer to the process of data extraction in a scoping review as data charting.
§ The process of systematically examining research evidence to assess its validity, results, and relevance before using it to inform a decision. This term is used for items 12 and 19 instead of "risk of bias" (which is more applicable to systematic reviews of interventions) to include and acknowledge the various sources of evidence that may be used in a scoping review (e.g., quantitative and/or qualitative research, expert opinion, and policy document).

From: Tricco AC, Lillie E, Zarin W, O'Brien KK, Colquhoun H, Levac D, et al. PRISMA Extension for Scoping Reviews (PRISMAScR): Checklist and Explanation. Ann Intern Med. 2018;169:467–473. doi: 10.7326/M18-0850.

For peer review only - http://bmjopen.bmj.com/site/about/guidelines.xhtml

Supplementary file 2: PRISMA 2009 Flow Diagram (adapted for a scoping review)

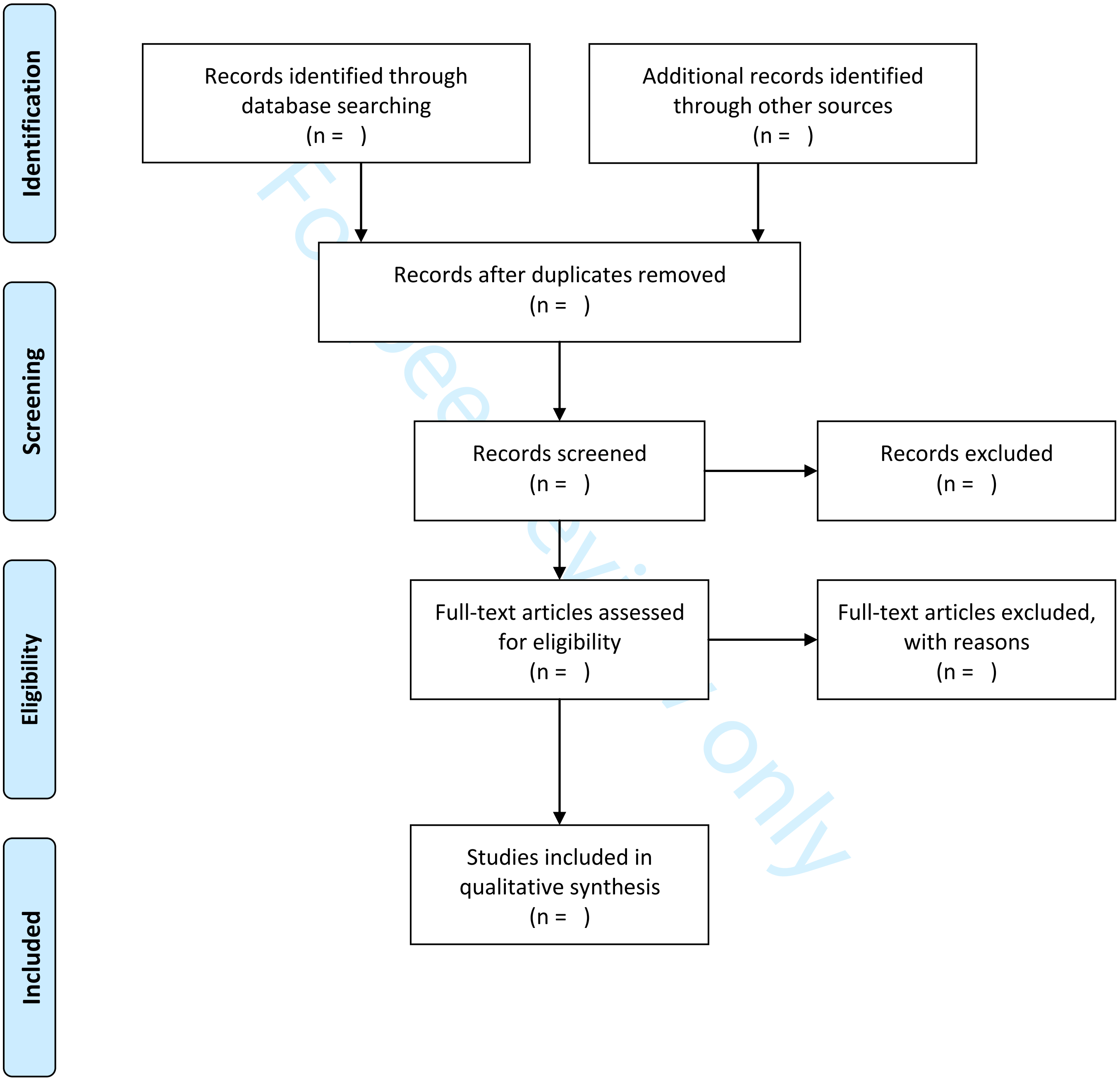

From: Moher D, Liberati A, Tetzlaff J, Altman DG, The PRISMA Group (2009). *P*referred *R*eporting *I*tems for *S*ystematic Reviews and *M*eta-*A*nalyses: The PRISMA Statement. PLoS Med 6(7): e1000097. doi:10.1371/journal.pmed1000097

For more information, visit www.prisma-statement.org.

Supplementary file 3

Search strategy to be conducted in CINAHL

(((MH "Midwifery+") OR (MH "Students, Nurse Midwifery") OR (MH "Students, Midwifery") OR (MH "Lay Midwifery") OR (MH "Research, Midwifery") OR (MH "Nurse-Midwifery Service") OR (MH "Nurse Midwifery") OR (MH "Midwifery Service+")) AND ((MH "Professional Knowledge+") OR (MH "Clinical Competence+") OR (MH "Professional Competence+") OR (MH "Competency Assessment") OR (MH "Competency Assessment") OR (MH "Education, Competency-Based") OR (MH "Teaching Methods+/EV") OR (MH "Critical Thinking/EV")) AND (impact OR evaluation OR tools OR measuring OR exam* OR test* OR impact*)) OR (MH "Education, Midwifery" AND (evaluation OR impact OR tools OR testing OR exam* OR competen* OR "critical thinking"))

For peer review only

Supplementary file 4

Data extraction form

Ref. ID	First Author	Year of Publication	Year(s) of Study Coverage	Study Design	Country	Study location	Study setting	Education area of focus	Cadres trained	On-site	Off-site	Online	Lectures	Simulation	Mentorship	Training Facilitator	Identifiable training materials	

For peer review only

For peer review only - http://bmjopen.bmj.com/site/about/guidelines.xhtml

Supplementary file 5

Tools to map education and training material content to ICM Competencies for Midwifery Practice and Lancet Series Quality Maternal and Newborn Care framework

Data collection tool: Mapping to ICM Essential Competencies for Midwifery Practice

**Reference ID**									
**First author**									
**Year of publication**									
**Identified training resource**									
**Category 1: General Competencies**									
**1a: Assume responsibility for own decisions and actions as an autonomous practitioner**									
Knowledge	Principles of accountability and transparency								
	Principles and concepts of autonomy								
	Principles of self-assessment and reflective practice								
	Personal beliefs and their influence on practice								
	Knowledge of evidence-based practices								
Skills and Behaviours	Demonstrate behaviour that upholds the public trust in the profession								
	Participate in self-evaluation, peer review and other quality improvement activities								
	Balance the responsibility of the midwife to provide best care with the autonomy of the woman to make her own decisions								
	Explain the midwife's role in providing care that is based on relevant laws, ethics and evidence.								
**1b: Assume responsibility for self-care and self-development as a midwife**									
Knowledge	Strategies for managing personal safety particularly within the facility or community setting								
Skills and behaviours	Display skills in management of self in relation to time management, uncertainty, change and coping with stress								
	Assume responsibility for personal safety in various practice settings								
	Maintain up-to-date skills and knowledge concerning protocols, guidelines and safe practice								
	Remain current in practice by participating in continuing professional education for example, participating in learning opportunities that apply evidence to practice to improve care such as mortality reviews or policy reviews)								
	Identify and address limitations in personal skill, knowledge, or experience								
	Promote the profession of midwifery, including participation in professional organizations at the local and national level								
**1c: Appropriately delegate aspects of care and provide supervision**									
Knowledge	Policies and regulation related to delegation								
	Supportive strategies to supervise others								
	Role of midwives as preceptors, mentors, supervisors, and role models								
Skills and behaviours	Provide supervision to ensure that practice is aligned with evidence-based clinical practice guidelines								
	Support the profession's growth through participation in midwifery education in the roles of clinical preceptor, mentor, and role model								
**1d: Use research to inform practice**									
Knowledge	Principles of research and evidence-based practice								
	Epidemiologic concepts relevant to maternal and infant health								
	Global recommendations for practice and their evidence base (e.g. World Health Organization guidelines)								
Skills and behaviours	Discuss research findings with women and colleagues								

For peer review only - http://bmjopen.bmj.com/site/about/guidelines.xhtml

Supplementary file 5
Tools to map education and training material content to ICM Competencies for Midwifery Practice and Lancet Series Quality Maternal and Newborn Care framework

	Support research in midwifery by participating in the conduct of research								
**1e: Uphold fundamental human rights of individuals when providing midwifery care**									
Knowledge	Laws and/or codes that protect human rights								
	Sexual, reproductive health rights of women and girls								
	Development of gender identity and sexual orientation								
	Principles of ethics and Human Rights within midwifery practice								
Skills and behaviours	Provide information to women about their sexual and reproductive health rights								
	Inform women about the scope of midwifery practice and women's rights and responsibilities								
	Provide information and support to individuals in complex situations where there are competing ethical principles and rights								
	Practice in accordance with philosophy and code of ethics of the ICM and national standards for health professionals								
	Provide gender sensitive care								
**1f: Adhere to jurisdictional laws, regulatory requirements, and codes of conduct for midwifery practice.**									
Knowledge	The laws and regulations of the jurisdiction regarding midwifery								
	National/state/local community standards of midwifery practice								
	Ethical principles								
	ICM and other midwifery philosophies, values, codes of ethics								
Skills and behaviours	Practise according to legal requirements and ethical principles								
	Meet requirements for maintenance of midwifery registration								
	Protect confidentiality of oral information and written records about care of women and infants								
	Maintain records of care in the manner required by the health authority								
	Comply with all local reporting regulations for birth and death registration								
	Recognize violations of laws, regulations, and ethical codes and take appropriate action								
	Report and document incidents and adverse outcomes as required while providing care								
**1g: Facilitate women to make individual choices about care**									
Knowledge	Cultural norms and practices surrounding sexuality, sexual practices, marriage, the childbearing continuum, and parenting								
	Principles of empowerment								
	Methods of conveying health information to individuals, groups, communities								
Skills and behaviours	Advocate for and support women to be the central decision makers in their care								
	Assist women to identify their needs, knowledge, skills, feelings, and preferences throughout the course of care								
	Provide information and anticipatory guidance about sexual and reproductive health to assist women's decision making								
	Collaborate with women in developing a comprehensive plan of care that respects her preferences and decisions								
**1h: Demonstrate effective interpersonal communication with women and families, health care teams, and community groups**									
Knowledge	Role and responsibilities of midwives and other maternal – infant health providers								
	Principles of effective communication								
	Principles of effectively working in health care teams								
	Cultural practices and beliefs related to childbearing and reproductive health								
	Principles of communication in crisis situations, e.g. grief and loss, emergencies								
Skills and behaviours	Listen to others in an unbiased and empathetic manner								

For peer review only - http://bmjopen.bmj.com/site/about/guidelines.xhtml

Supplementary file 5

Tools to map education and training material content to ICM Competencies for Midwifery Practice and Lancet Series Quality Maternal and Newborn Care framework

	Respect one others' point of view								
	Promote the expression of diverse opinions and perspectives								
	Use the preferred language of the woman or an interpreter to maximise communication								
	Establish ethical and culturally-appropriate boundaries between professional and non-professional relationships								
	Demonstrate cultural sensitivity to women, families, and communities								
	Demonstrate sensitivity and empathy for bereaved women and family members								
	Facilitate teamwork and inter-professional care with other care providers (including students) and community groups/ agencies								
	Establish and maintain collaborative relationships with individuals, agencies, institutions that are part of referral networks								
	Convey information accurately and clearly and respond to the needs of individuals								
**1i: Facilitate normal birth processes in institutional and community settings, including women's homes**									
Knowledge	Normal biologic, psychologic, social, and cultural aspects of reproduction and early life								
	Practices that facilitate and those that interfere with normal processes								
	Policies and protocols about care of women in institutional and community settings								
	Availability of resources in various settings								
	Community views about and utilization of health care facilities and place(s) of birth								
Skills and behaviours	Promote policies and a work culture that values normal birth processes								
	Utilize human and clinical care resources to provide personalized care for women and their infants								
	Provide continuity of care by midwives known to woman								
**1j: Assess the health status, screen for health risks, and promote general health and well-being of women and infants**									
Knowledge	Health needs of women related to reproduction								
	Health conditions that pose risks during reproduction								
	Health needs of infants and common risks								
Skills and behaviours	Conduct a comprehensive assessment of sexual and reproductive health needs								
	Assess risk factors and at-risk behaviour								
	Order, perform, and interpret laboratory and/ or imaging screening tests								
	Exhibit critical thinking and clinical reasoning informed by evidence when promoting health and well being								
	Provide health information and advice tailored to individual circumstances of women and their families								
	Collaborate with women to develop and implement a plan of care								
**1k: Prevent and treat common health problem related to reproduction and early life**									
Knowledge	Common health problems related to sexuality and reproduction								
	Common health problems and deviations from normal of newborn infants								
	Treatment of common health problems								
	Strategies to prevent and control the acquisition and transmission of environmental and communicable diseases								
Skills and behaviours	Maintain/promote safe and hygienic conditions for women and infants								
	Use universal precautions consistently								
	Provide options to women for coping with and treating common health problems								
	Use technology and interventions appropriately to promote health and prevent secondary complications								
	Recognize when consultation or referral is indicated for managing identified health problems, including consultation with other midwives								

For peer review only - http://bmjopen.bmj.com/site/about/guidelines.xhtml

Supplementary file 5

Tools to map education and training material content to ICM Competencies for Midwifery Practice and Lancet Series Quality Maternal and Newborn Care framework

	Include woman in decision-making about referral to other providers and services								
**1l: Recognise abnormalities and complications and institute appropriate treatment and referral**									
Knowledge	Complications/pathologic conditions related to health status								
	Emergency interventions/life-saving therapies								
	Limits of midwifery scope of practice and own experience								
	Available referral systems to access medical and other personnel to manage complications								
	Community/facility plans and protocols for accessing resources in timely manner								
Skills and behaviours	Maintain up-to-date knowledge, life-saving skills, and equipment for responding to emergency situations								
	Recognize situations requiring expertise beyond midwifery care								
	Maintain communication with women about nature of problem, actions taken, and referral if indicated								
	Determine the need for immediate intervention and respond appropriately								
	Implement timely and appropriate intervention, inter-professional consultation and/or timely referral taking account of local circumstances								
	Provide accurate oral and written information to other care providers when referral is made								
	Collaborate with decision-making if possible and appropriate								
**1m: Care for women who experience physical and sexual violence and abuse**									
Knowledge	Socio-cultural, behavioural, and economic conditions that often accompany violence and abuse								
	Resources in community to assist women and children								
	Risks of disclosure								
Skills and behaviours	Protect privacy and confidentiality								
	Provide information to all women about sources of help regardless of whether there is disclosure about violence								
	Inquire routinely about safety at home, at work								
	Recognize potential signs of abuse from physical appearance, emotional affect, related risk behaviours such as substance abuse								
	Provide special support for adolescents and victims of gender-based violence including rape								
	Refer to community resources, assist in locating safe setting as needed								
**Category 3: Care during labour and birth**									
**3a: Promote physiological labour and birth**									
Knowledge	Anatomy of maternal pelvis and fetus; mechanisms of labour for different fetal presentations								
	Physiologic onset and progression of labour								
	Evidence informed intrapartum care policies and guidelines, including avoidance of routine interventions in normal labour and birth								
	Cultural and social beliefs and traditions about birth								
	Signs and behaviours of labour progress; factors that impede labour progress								
	Methods of assessing fetus during labour								
Skills and behaviours	Provide care for a woman in the birth setting of her choice, following policies and protocols								
	Obtain relevant obstetric and medical history								
	Perform and interpret focused physical examination of the woman and fetus								
	Order and interpret laboratory tests if needed								
	Assess woman's physical and behavioural responses to labour								
	Provide information, support, and encouragement to woman and support persons throughout labour and birth								

For peer review only - http://bmjopen.bmj.com/site/about/guidelines.xhtml

Supplementary file 5

Tools to map education and training material content to ICM Competencies for Midwifery Practice and Lancet Series Quality Maternal and Newborn Care framework

	Provide respectful one-to-one care								
	Encourage freedom of movement and upright positions								
	Provide nourishment and fluids								
	Offer and support woman to use strategies for coping with labour pain, e.g. controlled breathing, water immersion, relaxation, massage, and pharmacologic modalities when needed								
	Assess regularly parameters of maternal-fetal status, and e.g. vital signs, contractions, cervical changes, and fetal descent								
	Use labour progress graphic display to record findings and assist in detecting complications, e.g. labour delay, fetal compromise, maternal exhaustion, hypertension, infection								
	Augment uterine contractility judiciously using non-pharmacological or pharmacological agents to prevent non-progressive labour								
	Prevent unnecessary routine interventions, e.g. amniotomy, electronic fetal monitoring, directed closed glottis pushing, episiotomy								
**3b: Manage a safe spontaneous vaginal birth; prevent, detect and stabilize complications**									
Knowledge	Manage a safe spontaneous vaginal birth								
	Evidence about conduct of third stage, including use of uterotonics								
	Potential complications and their immediate treatment e.g. shoulder dystocia,and excessive bleeding, fetal compromise, eclampsia, retained placenta								
	Management of emergencies as covered in emergency skills trainingprogrammes such as BEmONC,HMS								
	Signs of placental separation; appearance of normal placenta, membranes,and umbilical cord								
	Types of perineal and vaginal trauma requiring repair and suturing techniques								
Skills and behaviours	Support the woman to give birth in her position of choice								
	Ensure clean environment, presence of clean necessary supplies and sourceof warmth								
	Coach woman about pushing to control expulsion of presenting part, avoid routine episiotomy								
	Undertake appropriate manoeuvers and use maternal position to facilitatevertex, face, or breech birth								
	Expedite birth in presence of fetal distress								
	Delay cord clamping								
	Manage nuchal cord								
	Assess immediate condition of newborn								
	Provide skin to skin contact and warm environment								
	Deliver placenta and membranes and inspect for completeness								
	Assess uterine tone, maintain firm contraction, and estimate and record maternal blood loss; manage excessive blood loss including administration of uterotonics								
	Inspect vaginal and perineal areas for trauma, and repair as needed,following policies and protocols								
	Provide first line measures to treat or stabilise identified conditions								
	Refer for continuing treatment of any complications as needed								
**3c: Provide care of the newborn immediately after birth**									
Knowledge	Normal transition to extra-uterine environment								
	Scoring systems to assess newborn status								

For peer review only

Supplementary file 5

Tools to map education and training material content to ICM Competencies for Midwifery Practice and Lancet Series Quality Maternal and Newborn Care framework

	Signs indicating need for immediate actions to assist transition								
	Interventions to establish breathing and circulation as covered in training programs such as HBS								
	Appearance and behaviour of healthy newborn infant								
	Method of assessing gestational age of newborn infant								
	Needs of small for gestational age and low birth weight infants								
Skills and behaviour	Use standardized method to assess newborn condition in the first minutes of life (Apgar or other); refer if needed								
	Institute actions to establish and support breathing and oxygenation, refer for continuing treatment as needed								
	Provide a safe warm environment for initiating breastfeeding and attachment (bonding) in the first hour of life								
	Conduct a complete physical examination of newborn in presence of mother/family; explain findings and expected changes e.g. colour of extremities, moulding of head. Refer for abnormal findings.								
	Institute newborn prophylaxis e.g. ophthalmic infection, and hemorrhagic disease, according to policies and guidelines								
	Promote care by mother, frequent feeding and close observation								
	Involve partner/support persons in providing newborn care								

For peer review only - http://bmjopen.bmj.com/site/about/guidelines.xhtml

Supplementary file 5

Tools to map education and training material content to ICM Competencies for Midwifery Practice and Lancet Series Quality Maternal and Newborn Care framework

Data collection tool: Mapping to Lancet Series Quality Maternal and Newborn Care framework

**Reference ID**									
**First author**									
**Year of publication**									
**Identified training resource**									
**Practice Categories**									
For all childbearing women and infants	Education								
	Information								
	Health Promotion								
	Assessment								
	Screening								
	Care planning								
	Promotion of normal processes, prevention of complications								
For childbearing women and infants with complications	First line management of complications								
	Medical obstetric neonatal services								
**Organisation of care**									
For all childbearing women and infants AND for all childbearing women and infants with complications	Available								
	Accessible								
	Acceptable								
	Good quality services								
	Adequate resources								
	Competent workforce								
	Continuity								
	Services integrated across community and facilities								
**Values**									
For all childbearing women and infants AND for all childbearing women and infants with complications	Respect								
	Communication								
	Community knowledge								
	Understanding								
	Care tailored to women's circumstances and needs								
**Philosophy**									
For all childbearing women and infants AND for all childbearing women and infants with complications	Optimising biological, psychological, social and cultural processes								
	Strengthening women's capabilities								
	Expectant management, using interventions only when indicated								
**Care providers**									
For all childbearing women and infants AND for all childbearing women and infants with complications	Practitioners who combine clinical knowledge and skills with interpersonal and cultural competence								
	Division of roles and responsibility based on need, competencies and resources								

For peer review only

For peer review only - http://bmjopen.bmj.com/site/about/guidelines.xhtml
